# Probing the Role of CYP2 Enzymes in the Atropselective Metabolism of Polychlorinated Biphenyls Using Liver Microsomes from Transgenic Mouse Models

**DOI:** 10.1021/acs.chemrestox.2c00276

**Published:** 2022-12-06

**Authors:** Hans-Joachim Lehmler, Eric Uwimana, Laura E. Dean, Nataliia Kovalchuk, Qing-Yu Zhang, Xinxin Ding

**Affiliations:** † Interdisciplinary Graduate Program in Human Toxicology and Department of Occupational and Environmental Health, University of Iowa, Iowa City, Iowa 52242, United States; ‡ Department of Pharmacology and Toxicology, College of Pharmacy, University of Arizona, Tucson, Arizona 85721, United States

## Abstract

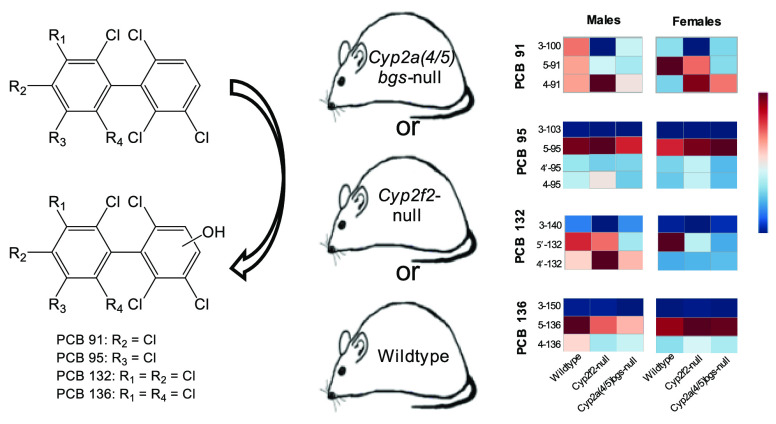

Chiral polychlorinated biphenyls (PCB) are environmentally relevant developmental neurotoxicants. Because their hydroxylated metabolites (OH-PCBs) are also neurotoxic, it is necessary to determine how PCB metabolism affects the developing brain, for example, in mouse models. Because the cytochrome P450 isoforms involved in the metabolism of chiral PCBs remain unexplored, we investigated the metabolism of PCB 91 (2,2′,3,4′,6-pentachlorobiphenyl), PCB 95 (2,2′,3,5′,6-pentachlorobiphenyl), PCB 132 (2,2′,3,3′,4,6′-hexachlorobiphenyl), and PCB 136 (2,2′,3,3′,6,6′-hexachlorobiphenyl) using liver microsomes from male and female *Cyp2a(4/5)bgs*-null, *Cyp2f2*-null, and wild-type mice. Microsomes, pooled by sex, were incubated with 50 μM PCB for 30 min, and the levels and enantiomeric fractions of the OH-PCBs were determined gas chromatographically. All four PCB congeners appear to be atropselectively metabolized by CYP2A(4/5)BGS and CYP2F2 enzymes in a congener- and sex-dependent manner. The OH-PCB metabolite profiles of PCB 91 and PCB 132, PCB congeners with one *para*-chlorine substituent, differed between null and wild-type mice. No differences in the metabolite profiles were observed for PCB 95 and PCB 136, PCB congeners without a *para*-chlorine group. These findings suggest that *Cyp2a(4/5)bgs*-null and *Cyp2f2*-null mice can be used to study how a loss of a specific metabolic function (e.g., deletion of *Cyp2a(4/5)bgs* or *Cyp2f2*) affects the toxicity of chiral PCB congeners.

## Introduction

Polychlorinated biphenyls (PCBs) are ubiquitous environmental pollutants. Although the intentional production of PCBs was banned decades ago, recent studies demonstrate that PCBs continue to be formed inadvertently by industrial processes.^1−3^ Because of their persistence in the environment, PCBs are detected in air, including indoor air in public schools,^2,4−6^ and foodstuffs.^7−9^ For example, several PCB congeners were detected in the serum of women enrolled in the Markers of Autism Risk in Babies–Learning Early Signs (MARBLES) study, an epidemiological study of pregnant women at risk of having a child with a neurodevelopmental disorder.^10^ Other studies report the presence of PCBs in post-mortem human brain tissue.^11−13^ Human exposure to PCBs occurs via several routes, including direct contact with consumer products^14^ and diet.^8,15^ Recent studies show that inhalation is an important and current route of PCB exposure.^16^ There is also evidence that PCB metabolites, such as OH-PCBs and PCB sulfates, are present in the brains of laboratory animals.^17,18^ Moreover, some lower OH-PCBs have recently been detected in post-mortem human brain samples.^13^

These findings raise environmental health concerns because PCBs and their metabolites are widely accepted developmental neurotoxicants that cause cognitive deficits.^19,20^ Laboratory studies implicate PCB congeners with multiple *ortho*-substituents and their hydroxylated metabolites in adverse neurotoxic outcomes.^21^ For example, both parent PCBs and their hydroxylated metabolites can be potent sensitizers of the ryanodine receptor (RyR).^22−24^ The endocrine-disrupting effects of hydroxylated PCBs have also been linked to effects on behavior and neurodevelopment in rats *in vivo*.^25,26^ Several of the RyR-active PCB congeners (*e.g.*, PCB 95) and their metabolites are chiral because they exist as rotational isomers that form nonsuperimposable mirror images of each other.^27,28^ Chiral PCB congeners atropselectively affect cellular targets, such as the RyR, implicated in PCB developmental neurotoxicity.^29−31^ Thus, biological processes resulting in the atropisomeric enrichment of PCBs or their metabolites, such as the atropselective metabolism of chiral PCBs to OH-PCBs, are expected to influence PCB toxicity.

Laboratory studies demonstrate species differences in the oxidation of neurotoxic PCBs, including chiral PCB congeners, by cytochrome P450 (P450) enzymes.^32−37^ For example, PCB 95 is primarily oxidized to 4′-OH-PCB 95 by human liver microsomes.^32^ In contrast, PCB 95 is preferentially metabolized to a *meta*-hydroxylated metabolite in rodents.^38−42^ Studies with recombinant enzymes demonstrate that rat CYP2B1 is involved in the oxidation of PCB 95 and structurally related chiral PCB congeners;^38,39,43^ however, the P450 isoforms involved in the metabolism of these PCB congeners in mice have not been identified because recombinant mouse enzymes are not commercially available. Studies of precision-cut liver tissue slices from mice pretreated with phenobarbital, a CYP2B inducer, indicate that CYP2B isoforms metabolize *ortho*-substituted PCB congeners in mice.^40^ Several human P450 isoforms, including CYP2A6 and CYP2B6, oxidize chiral PCB congeners in an isoform-dependent manner.^32−34^ In contrast, PCB congeners without *ortho*-chlorine substituents, including dioxin-like PCBs, are metabolized by CYP1A enzymes.^44^ Moreover, interindividual differences in the microsomal metabolism of chiral neurotoxic PCB congeners have been reported.^45−47^

The studies described above demonstrate that the oxidation of chiral PCBs by CYP2 enzymes is atropselective. However, it is unknown how the atropselective metabolism of PCBs affects PCB developmental neurotoxicity *in vivo*. Transgenic animal models are potential tools that can be used to investigate how specific P450 isoforms affect PCB developmental neurotoxicity *in vivo* by knocking out specific P450 isoforms or creating humanized mouse models of P450 enzymes. For example, studies with transgenic mouse strains revealed that genetic differences in CYP1A and CYP1B isoforms alter the disposition of dioxin-like PCBs and their metabolites and affect neurotoxic outcomes following developmental PCB exposure.^48,49^ Importantly, elevated maternal CYP1A2 levels in the liver protect the offspring from memory and learning deficits induced by dioxin-like PCBs.^50^ Mice with a liver-specific deletion of cytochrome P450 reductase (CPR-null mice), the obligate electron donor of P450 enzymes, have been used to study the disposition of PCBs and their metabolites.^51,52^ Steroid and xenobiotic receptor (SXR)-null mice have been used to assess the toxicity of PCB 153, a di-*ortho*-substituted PCB congener prevalent in human tissues.^53^ This study reported elevated hydroxylated PCB 153 metabolite levels in tissues from SXR-null mice, consistent with a protective role of SXR due to the induction of the expression of P450 isoforms regulated by SXR.

Several *Cyp2*-null mouse models, including *Cyp2a(4/5)bgs*-null and *Cyp2f2*-null mice, have been generated to probe the role of CYP2 enzymes in toxic outcomes. However, to date, the atropselective metabolism of neurotoxic PCB congeners, including PCB 91, PCB 95, PCB 132, and PCB 136, to neurotoxic OH-PCBs has not been investigated in these mouse models. To close this knowledge gap and demonstrate that these models can be used to study how CYP2-mediated metabolism affects toxic outcomes *in vivo*, the present study characterizes the atropselective metabolism of these environmentally relevant PCB congeners using pooled liver microsomes.

## Experimental Procedures

### Materials

**Figure 1:**
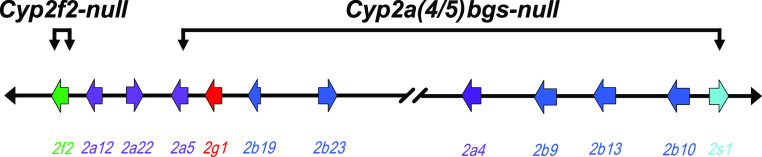
Mouse models used in this study. *Cyp2f2*- and *Cyp2a(4/5)bgs*-null mice were generated by deleting the indicated segments from the *Cyp2f2–2s1* cluster on mouse cytochrome 7, as described previously.^54−56^ The organization of this gene cluster was published previously.^74^

**Figure 2:**
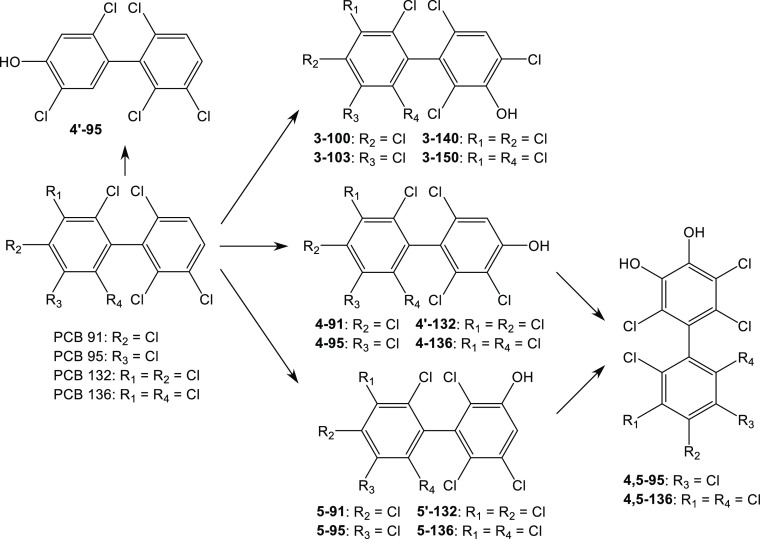
Simplified metabolism scheme showing the structures and abbreviations of mono- and dihydroxylated metabolites of PCB 91, PCB 95, PCB 132, and PCB 136 observed in metabolism studies with mouse liver microsomes. R_1_–R_4_ are H if not indicated otherwise.

Pooled liver microsomes were prepared from male and female *Cyp2a(4/5)bgs*-null, *Cyp2f2*-null, and wild-type mice. Details about the mouse models have been described previously.^54−56^ All mouse strains have a C57BL/6 background. Figure 1 illustrates the gene(s) knocked out in the respective null mice. Pooled liver microsomes from male and female C57BL/6 mice were purchased from Xenotech (Lenexa, Kansas) to assess the intraday and interday variability of the metabolism studies. The commercial sources or the synthesis and authentication of the PCB metabolite standards are summarized in an earlier publication;^32^ for the structures and abbreviations of the OH-PCB metabolites, see Figure 2 and the Supporting Information.

### Metabolism of PCBs by Mouse Liver Microsomes

*In vitro* PCB metabolism studies were conducted as described previously.^46,47,57^ Briefly, the incubation systems consisted of 0.1 mg/mL microsomal protein, 50 μM PCB, 1 mM NADPH, 0.1 M sodium phosphate buffer (pH 7.4), and 3 mM magnesium chloride in a final volume of 2 mL. The mixtures were preincubated for 5 min, then PCB 91, PCB 95, PCB 132, or PCB 136 (final concentration of 50 μM; DMSO ≤ 0.5% v/v) was added to start the reaction. The incubations lasted 30 min at 37 °C in a shaking water bath. All reactions were terminated by adding 2 mL of ice-cold sodium hydroxide (0.5 M) to the mixture, and the incubations were heated at 110 °C for 10 min. The formation of PCB metabolites was linear up to 30 min under these conditions, as demonstrated with pooled liver microsomes from male and female C57BL/6 mice (median *R*^2^ = 0.98 for all metabolites). These experimental conditions were selected to produce OH-PCB levels sufficient for robust atropselective analyses. If not stated otherwise, all incubations were performed in triplicate. Recovery standards PCB 117 (100 ng in 200 μL of isooctane) and 4′-159 (68 ng in 50 μL of methanol) were added to each sample and the reference standards. PCBs and their hydroxylated metabolites were extracted and derivatized as described earlier.^32^ Controls with phosphate buffer only, without NADPH, without microsomes, and without PCBs and controls with heat-inactivated microsomes were included with each experiment to ensure the rigor and reproducibility of the metabolism studies. No metabolites were detected in these control samples. Metabolism studies with racemic PCB 91 using pooled mouse liver microsomes demonstrate the intraday and interday reproducibility of the metabolism studies (Table S1).

### Quantification of Metabolites

The levels of hydroxylated PCB metabolites in the concentrated sample extracts were quantified on an Agilent 7890A gas chromatograph with a ^63^Ni microelectron capture detector (μECD) (Agilent, Santa Clara, California) and an SPB-1 capillary column (60 m length, 250 μm inner diameter, 0.25 μm film thickness; Supelco, St Louis, Missouri) using the internal standard method as described previously.^32^ OH-PCB levels, adjusted for the microsomal protein content, are presented in Table S2.

### Atropselective Analysis of PCBs and Their Metabolites

The chiral signatures of OH-PCBs, which were analyzed as the methylated derivatives, in sample extracts were determined using Agilent 6890 or Agilent 7890A gas chromatographs equipped with the following capillary columns (Tables S3 and S4): ChiralDex B-DM (BDM; Supelco) for PCB 91, 5-91, 4-91, PCB 95, 3-103, 4′-95, PCB 132, and 5′-132; Chirasil-Dex (CD; Agilent) for PCB 132, 5′-132, PCB 136, 3-150, 5-136, and 4-136; Cyclosil-B (CB; Agilent) for PCB 136, 3-150, and 4-136; and ChiralDex G-TA (GTA; Supelco) for 3-100 and 3′-140.^32^ Atropisomers of 4′-132 could not be resolved on any of the columns used.^58^ The helium flow was 3 mL/min. The injectors and detectors were maintained at 250 °C. Temperature programs were as previously described.^32^

The enantiomeric fractions (EFs), resolutions, and retention times of the analytical standards are summarized in Table S4, and representative chromatograms are shown in Figures S1–S6. The atropisomers are identified based on their elution order on the gas chromatographic column. The EF values of atropisomers were calculated by the drop valley method as EF = area E_1_/(area E_1_ + area E_2_), where area E_1_ and area E_2_ denote the peak areas of the first- (E_1_) and second-eluting (E_2_) atropisomers,^59^ respectively, to facilitate a comparison with our earlier studies.^32,40,46,47,57^

### Quality Assurance and Quality Control

The limits of detection (LOD) of the PCB metabolites were calculated from blank buffer samples as LOD = mean blanks + *k* × standard deviation blanks (*k* is the Student’s *t* value for a degree of freedom *n* – 1 = 11 at a 99% confidence level), and values are summarized in Table S5. The recoveries of PCB 117 and 4′-159 were 87 ± 7% (range from 63 to 112, relative standard deviation of 8%, *n* = 171) and 105 ± 7% (range from 82 to 126, relative standard deviation of 7%, and *n* = 170), respectively. Levels of PCB and its metabolites were not adjusted for recovery to facilitate a comparison with earlier studies.^47,60^ The relative retention times (RRTs) of the metabolites, calculated relative to PCB 204 as the internal standard, were within 0.5% of the RRT of the respective authentic standard. No OH-PCBs were detected in control incubations.

### Data Analysis

PCB metabolite profiles were compared using the similarity coefficient cos θ (Table S6).^61^ cos θ ranges from 0 to 1, where a value of 0 indicates completely different profiles and a value of 1 indicates identical profiles. OH-PCB levels are expressed as the mean ± standard deviation. Statistically significant differences in the levels of OH-PCB metabolites formed by mouse microsomal preparations were examined using Student’s *t* test or with two-way analysis of variance (ANOVA), followed by Bonferroni post-test, using GraphPad Prism (GraphPad Software, San Diego, California).

## Results and Discussion

### Oxidation of PCBs by *Cyp2a(4/5)bgs*-null and *Cyp2f2*-null vs Wild-Type Mouse Liver Microsomes

P450 enzymes can oxidize PCBs to OH-PCBs; however, the P450 isoforms involved in PCB metabolism in mice and many other mammalian species are frequently unknown. Here, we probe the metabolism of four chiral PCB congeners using liver microsomes from *Cyp2a(4/5)bgs*-null and *Cyp2f2*-null mice. The following sections discuss OH-PCB metabolites formed by the different microsome preparations and evaluate differences in the total OH-PCB levels, the OH-PCB profiles, and the levels of individual OH-PCB congeners in metabolism studies with liver microsomes from male and female wild-type, *Cyp2a(4/5)bgs*-null and *Cyp2f2*-null mice for PCB 91, PCB 95, PCB 132, and PCB 136. We expect that the knockout of CYP2 enzymes forming specific OH-PCB congeners affects the OH-PCB profiles and results in lower OH-PCB levels in microsomal metabolism studies with microsomes from *Cyp2a(4/5)bgs*-null and *Cyp2f2*-null mice than wild-type mice.

### Identification of PCB 91 Metabolites in Wild-Type, *Cyp2f2*-null, and *Cyp2a(4/5)bgs*-null Mice

PCB 91 was oxidized by P450 enzymes to 3-100 (1,2-shift product), 5-91, and 4-91 in microsomal preparations from male and female wild-type mice, *Cyp2f2*-null mice, and *Cyp2a(4/5)bgs*-null mice (Figure 2). The formation of 5-91 and 4-91 is noteworthy because both OH-PCB congeners are RyR-active.^22^ The same OH-PCB 91 metabolites were detected in studies with precision-cut liver slices from female mice pretreated with phenobarbital, an inducer of CYP2B enzymes,^40^ and in mice *in vivo*.^62^ The formation of 3-100, 5-91, and 4-91 has also been reported in metabolism studies with recombinant human P450 enzymes (*i.e.*, CYP2A6, CYP2B6, and CYP2E1), rat CYP2B1, and human liver microsomes.^32,38,46^ 3-100, 5-91, and 4-91 were also detected in the blood, liver, and feces of mice exposed orally to PCB 91, with 5-91 being the major metabolite formed *in vivo*.^62^

Although the same PCB 91 metabolites formed in different model systems, the rank order of the metabolites differed between model systems, species, and P450 isoforms (Table S2). In the present study, 5-91 was the major metabolite detected in studies with female wild-type mouse liver microsomes. Similarly, 5-91 was the major PCB 91 metabolite formed in precision-cut liver tissue slices from phenobarbital-pretreated female mice.^40^ 5-91 was also the major OH-PCB metabolite observed in studies with recombinant CYP2B1 and CYP2B6, whereas 3-100 was the major metabolite formed from CYP2A6 and in human liver microsomes.^32,38,46^ In the present study, microsomes from male wild-type and transgenic mice showed a distinctively different rank order of the metabolites analyzed. Briefly, PCB 91 metabolite levels followed the order 4-91 > 5-91 > 3-100 in experiments with microsomes from male and female *Cyp2f2*-null mice. Similarly, 4-91 was the major metabolite formed by female *Cyp2a(4/5)bgs*-null mouse microsomes, followed by 5-91 and 3-100. PCB 91 metabolite levels followed the rank order of 4-91 ∼ 5-91 ∼ 3-100 in incubations with mouse microsomes from male wild-type and *Cyp2a(4/5)bgs*-null mice.

### Comparison of Total PCB 91 Metabolite Levels Across Genotypes and Sex

**Figure 3:**
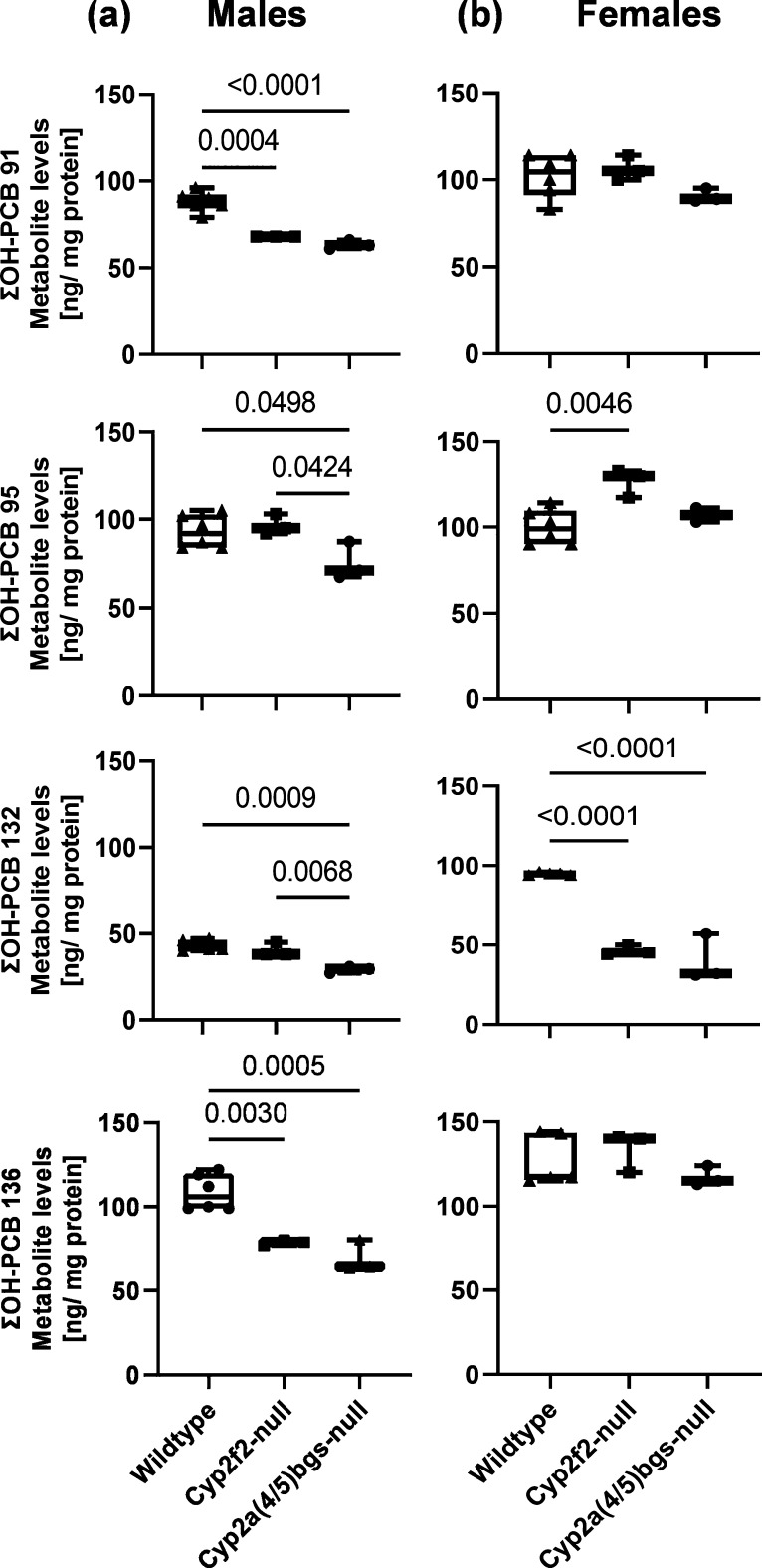
Differences in the total OH-PCB (ΣOH-PCB) metabolite profiles are due to genotype-dependent differences in the levels of individual PCB metabolites in experiments with pooled liver microsomes from (A) male and (B) female mice. The data were expressed as ng/mg protein. Metabolite levels were compared using one-way ANOVA.

We compared the total OH-PCB levels (ΣOH-PCB) to identify genotype and sex-dependent differences in the metabolism of PCB 91 (Figure 3). ΣOH-PCB levels were lower in studies that used microsomes from male *Cyp2f2*-null and *Cyp2a(4/5)bgs*-null mice rather than wild-type mice. This result indicates the role of CYP2F2 and CYP2A(4/5)BGS enzymes in the metabolism of PCB 91. In contrast, no significant differences in ΣOH-PCB levels by genotype were observed in experiments using microsomes from female mice. ΣOH-PCB levels were significantly higher in incubations with microsomes from female mice that those with male mice for each genotype. These sex differences were more pronounced for incubations with microsomes from *Cyp2f2*-null and *Cyp2a(4/5)bgs*-null mice than those with wild-type mice. *In vivo* studies are needed to determine if these differences translate into sex differences in the elimination of PCB 91, especially in the two null mouse strains.

### Comparison of PCB 91 Metabolite Profiles Across Genotypes and Sex

**Figure 4:**
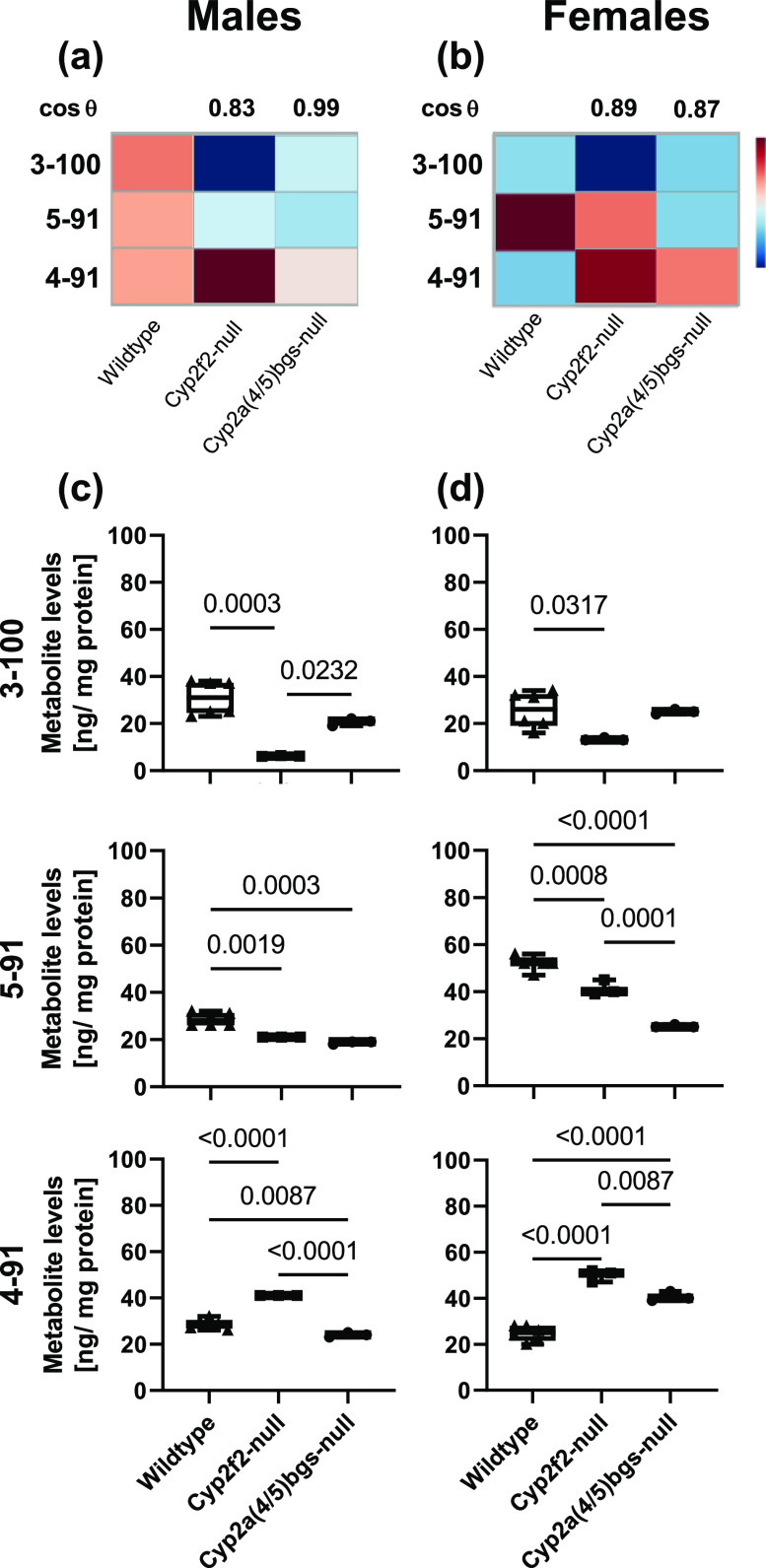
A heatmap-type comparison of the metabolite profiles formed from PCB 91 in incubations with pooled liver microsomes from (A) male and (B) female *Cyp2a(4/5)bgs*-null, *Cyp2f2*-null, and the corresponding wild-type mice reveals differences in the hydroxylated PCB metabolite levels across genotypes. These differences in the metabolite profiles are due to genotype-dependent differences in the levels of individual PCB 91 metabolites in experiments with pooled liver microsomes from (C) male and (D) female mice; significant changes are noted by *p*-values in the figures. The data are expressed as ng/mg protein and, in panels A and B, are visualized using the heatmap function implemented by Metabolanalyst. Metabolite levels were compared using one-way ANOVA. 3-100, 2,2′,4,4′,6-pentachlorobiphenyl-3-ol; 5-91, 2,2′,3,4′,6-pentachlorobiphenyl-5-ol; and 4-91, 2,2′,3,4′,6-pentachlorobiphenyl-4-ol.

A comparison of the OH-PCB metabolite profiles also showed genotype- and sex-dependent differences (Figure 4a and b). Interestingly, the metabolite levels differed between *Cyp2f2*-null and wild-type mouse microsomal incubation. These differences were more pronounced in male microsomal preparations than female microsomal preparations (cos θ = 0.83 and 0.89, respectively). We also observed differences in the metabolite profile in experiments using microsomes from female *Cyp2a(4/5)bgs*-null mice vs wild-type mice. In contrast, microsomal preparations of male *Cyp2a(4/5)bgs*-null mice and wild-type mice formed identical metabolite profiles. As with the ΣOH-PCB levels, these differences suggest a sex-dependent role of CYP2F2 and CYP2A(4/5)BGS enzymes in the metabolism of PCB 91. The OH-PCB metabolite profile showed small sex differences across all three genotypes, with cos θ ranging from 0.90 for *Cyp2f2*-null mouse microsomes to 0.96 for *Cyp2a(4/5)bgs*-null mouse microsomes. Sex differences in the metabolism of PCB 91 in mice have not been reported to date.

### Genotype-Dependent Formation of Individual PCB 91 Metabolites

Both male and female *Cyp2f2*-null mouse microsomes generated significantly lower levels of 3-100 compared to male wild-type mice (Figure 4c and d). 3-100 levels were also lower in incubations with *Cyp2f2*-null mouse microsomes than those with *Cyp2a(4/5)bgs*-null mouse microsomes. This difference reached statistical significance only for microsomes from male mice. Levels of 3-100 did not differ when comparing wild-type and *Cyp2a(4/5)bgs*-null mouse microsomes. Microsomes from male and female *Cyp2f2*-null and *Cyp2a(4/5)bgs*-null mice formed significantly lower levels of 5-91 compared to those from male wild-type mice (Figure 4c and d). *Cyp2a(4/5)bgs*-null mouse microsomes formed significantly lower levels of 5-91 than the corresponding *Cyp2f2*-null mouse microsomes in experiments with female microsomes. Finally, 4-91 levels were significantly lower in experiments with male and female *Cyp2a(4/5)bgs*-null mice microsomes than those with *Cyp2f2*-null mouse microsomes (Figure 4c amd d). Moreover, 4-91 levels were significantly lower in experiments with male *Cyp2a(4/5)bgs*-null mouse microsomes than those with wild-type mouse microsomes. In contrast, the levels of 4-91 formed by wild-type microsomes were lower compared to studies using microsomes from male and female *Cyp2f2*-null and female *Cyp2a(4/5)bgs*-null mice. Overall, these results suggest that CYP2F2 is involved in forming 3-100 and 5-91 from PCB 91 in male and female mice. Moreover, CYP2A(4/5) BGS enzymes may play a role in forming 4-91 in male mice.

### Identification of PCB 95 Metabolites in Wild-Type, *Cyp2f2*-null, and *Cyp2a(4/5)bgs*-null Mice

PCB 95 was metabolized to 3-103 (1,2-shift product), 5-95, 4′-95, and 4-95 by all microsomal preparations investigated (Figure 2). 3′-95 and other metabolites were likely also formed; however, we could not identify these metabolites because authentic standards were unavailable. These OH-PCB metabolites were also formed by precision-cut liver tissue slices from female mice pretreated with phenobarbital or recombinant rat CYP2B1.^38,40^ The formation of these PCB 95 metabolites has also been observed in studies with pooled and single-donor human liver microsomes,^47^ and those using recombinant cytochrome P450 enzymes, including CYP2A6 and, to a lesser extent, CYP2B6 and CYP2E1.^32^ Similarly, studies in rats and mice detected these OH-PCBs in different compartments *in vivo*.^47,63,64^ The formation of 4-95 and 5-95 is of toxicological interest because both OH-PCB congeners are ligands for the RyR,^22^ a key player in the developmental neurotoxicity of PCBs.^21^

5-95 was the major metabolite formed by all microsomal preparations, followed by 4-95 and 4′-95 (Table S2). The 1,2-shift metabolite, namely, 3-103, was a minor metabolite. Similarly, 5-95 was the major metabolite formed by precision-cut liver tissue slices from female mice pretreated with phenobarbital;^40^ however, 5-95, 4′-95, and 4-95 are major monohydroxylated metabolites detected in tissue from PCB 95-exposed mice.^18,63−65^ Rat recombinant CYP2B1 preferentially formed 5-95.^38^ Tissue levels of 5-95, 4′-95, and 4-95 in whole blood and livers from male Wistar rats were comparable.^66^ In human model systems, 4′-95 was the major metabolite of PCB 95 detected in studies with pooled and single-donor human liver microsomes, followed by 3-103 and 4-95.^47^ 5-95 was a relatively minor metabolite in these studies. 4′-95 is primarily formed by human CYP2A6, whereas CYP2B6 and CYP2E1 form only low levels of PCB 95 metabolites.^32^ CYP2A6 also oxidizes other structurally similar PCB congeners in the *para*-position.^33,34^ These comparisons reveal distinct differences in the rank order of the OH-PCB 95 metabolites formed in mice compared to that in human models.

### Comparison of Total PCB 95 Metabolite Levels and Profiles Across Genotypes and Sex

We observed some genotype- and sex-dependent differences in the metabolism of PCB 95, similar to our results with PCB 91 (Figure 3). Briefly, ΣOH-PCB 95 levels were significantly lower in studies using male *Cyp2a(4/5)bgs*-null mouse microsomes than those using wild-type and *Cyp2f2*-null mouse microsomes, consistent with a contribution of CYP2A(4/5)BGS enzymes to the oxidation of PCB 95. In contrast, the ΣOH-PCB levels were significantly higher in experiments with female *Cyp2f2*-null mouse microsomes than those with female wild-type mouse microsomes. No clear role of CYP2F2 or CYP2A(4/5)BGS enzymes was identified in studies with microsomes from female mice.

**Figure 5:**
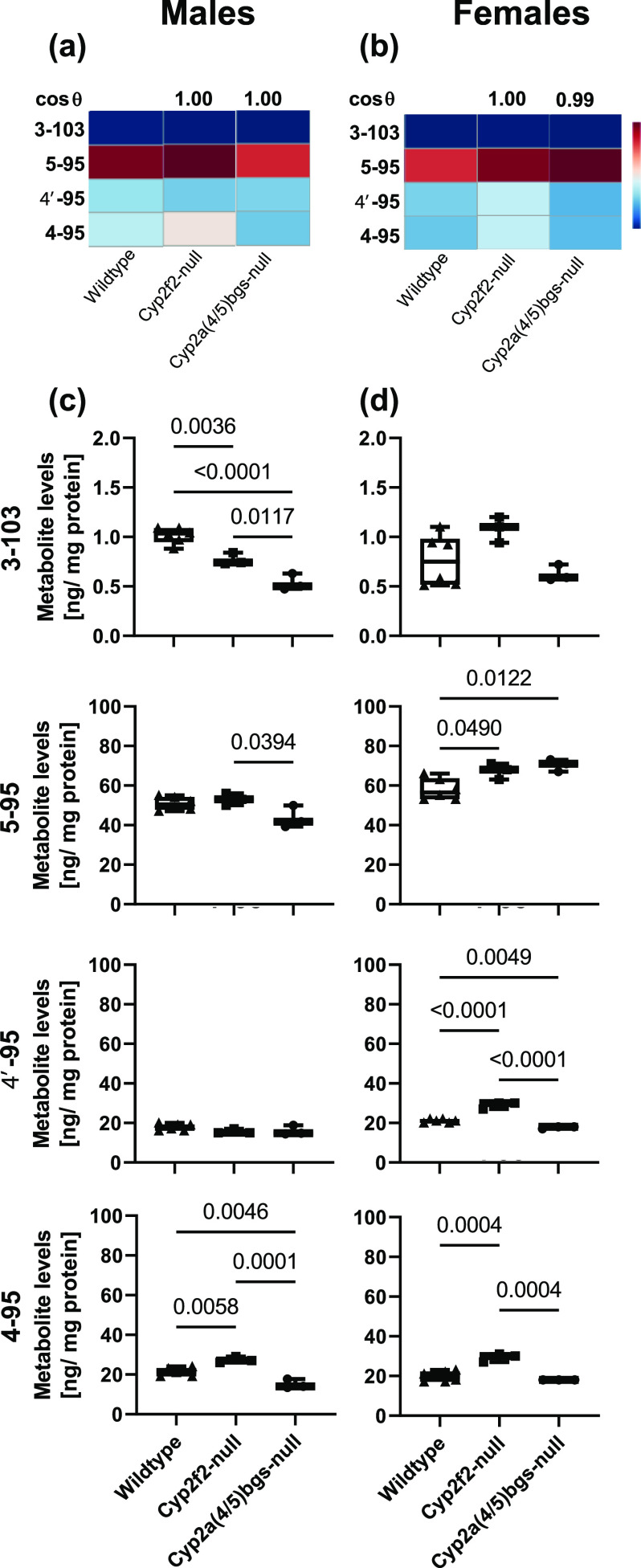
A heatmap-type comparison of the metabolite profiles formed from PCB 95 in incubations with pooled liver microsomes from (A) male and (B) female *Cyp2a(4/5)bgs*-null, *Cyp2f2*-null, and the corresponding wild-type mice reveals differences in the hydroxylated PCB metabolite levels across genotypes. These differences in the metabolite profiles are due to genotype-dependent differences in the levels of individual PCB 95 metabolites in experiments with pooled liver microsomes from (C) male and (D) female mice; significant changes are noted by *p*-values in the figures. The data are expressed as ng/mg protein and, in panels A and B, are visualized using the heatmap function implemented by Metabolanalyst. Metabolite levels were compared using one-way ANOVA. 3-103, 2,2′,4,5′,6-pentachlorobiphenyl-3-ol; 5-95, 2,2′,3,5′,6-pentachlorobiphenyl-5-ol; 4′-95, 2,2′,3,5′,6-pentachlorobiphenyl-4-ol; and 4-95, 2,2′,3,5′,6-pentachlorobiphenyl-4-ol.

Like PCB 91, the ΣOH-PCB levels were higher in incubations using female liver microsomes than those using male liver microsomes (Table S2). This sex difference was significantly more pronounced for microsomal preparations from *Cyp2f2*-null and *Cyp2a(4/5)bgs*-null mice, with 1.3- and 1.4-fold differences, respectively. Sex differences in PCB 95 metabolism in mice have not been reported previously and likely reflect higher levels of P450 isoforms involved in the metabolism of PCB 95 in the microsomal preparations investigated. Additional studies are needed to confirm that there are indeed sex-dependent differences in the expression of hepatic P450 enzymes and that these differences result in sex-dependent differences in the toxicokinetics of PCB 95. Despite these genotype and sex differences in the ΣOH-PCB levels, the OH-PCB 95 metabolite profiles were nearly identical when comparing incubations with the null mouse microsomes with wild-type microsomes, with cos θ ≥ 0.99 (Figure 5a and b). Moreover, in contrast to PCB 91, no sex differences were observed in the similarity coefficient for all three genotypes (cos θ ≥ 0.99).

### Genotype-Dependent Formation of Individual PCB 95 Metabolites

The 3-103 levels formed by the microsomal preparations from male mice significantly decreased in the order wild-type > *Cyp2f2*-null > *Cyp2a(4/5)bgs*-null (Figure 5c and d). No significant differences in 3-103 levels were observed in analogous experiments with microsomes from female mice. 5-95 levels were slightly lower in studies using microsomes from male *Cyp2a(4/5)bgs*-null mice than those using microsomes from *Cyp2f2*-null mice. In contrast, female *Cyp2f2*-null and *Cyp2a(4/5)bgs*-null mouse microsomes formed higher levels of this metabolite than wild-type mouse microsomes. 4′-95 levels were significantly higher in studies using female *Cyp2f2*-null mouse microsomes than those using female wild-type or *Cyp2a(4/5)bgs*-null mouse microsomes. For females, *Cyp2a(4/5)bgs*-null mouse microsomes formed significantly lower levels of 4′-95 than wild-type mouse microsomes. 4-95 levels were significantly higher in studies using male and female *Cyp2f2*-null mouse microsomes than in the corresponding studies using wild-type or *Cyp2a(4/5)bgs*-null mouse microsomes. For males, *Cyp2a(4/5)bgs*-null mouse microsomes formed significantly lower 4-95 levels than wild-type mouse microsomes. Overall, the mouse-strain-dependent differences of the OH-PCB 95 metabolite levels indicate the role of CYP2A(4/5)BGS but not CYP2F2 in forming several PCB 95 metabolites in the male mouse liver. This interpretation agrees with the trends observed for the ΣOH-PCB 95 (Figure 3).

### Identification of PCB 132 Metabolites in Wild-Type, *Cyp2f2*-null, and *Cyp2a(4/5)bgs*-null Mice

PCB 132 was metabolized to 3′-140 (1,2-shift product), 5′-132, and 4′-132 by all microsomal preparations investigated (Figure 2). Studies with precision-cut liver tissue slices from mice pretreated with the CYP2B inducer phenobarbital also reported the formation of these three hydroxylated metabolites.^40^ 3′-140, 5′-132, and 4′-132 were formed by some rat and human recombinant enzymes and pooled and single-donor human liver microsomes.^32,38,45^ The levels of these PCB 132 metabolites have not been reported *in vivo*, including in human samples. Based on disposition studies with PCB 91, PCB 95, and PCB 136 and limited mechanistic studies, we posit that the PCB 132 metabolites observed *in vitro* are also formed *in vivo* and are also developmental neurotoxicants.^22,45^

The rank order in which the different PCB 132 metabolites were formed depended on the microsomal preparation (Table S2). Wild-type mouse microsomes, irrespective of sex, generated 5′-132 as the major metabolite. Across both sexes, the 1,2-shift metabolite, namely, 3′-140, was a minor metabolite. 5′-132 was also the major metabolite detected in female *Cyp2f2*-null mouse preparations. Consistent with the observation that 5′-132 is the major metabolite formed in the wild-type mouse liver, 5′-132 was the major metabolite detected in metabolism studies using precision-cut liver tissue slices prepared from female mice exposed to phenobarbital, an inducer of CYP2B enzymes.^40^ Analogous to PCB 91, 5′-132 was the major metabolite formed by recombinant rat CYP2B1 and human CYP2B6.^32,38^ Distinct differences were observed in studies using human liver microsomes, where PCB 132 was oxidized preferentially in the *meta*-position to 5′-132 and 3′-140,^45^ or CYP2A6, where 3′-140 was the major metabolite.^32^ In contrast, male *Cyp2f2*-null and male and female *Cyp2a(4/5)bgs*-null mouse microsomes yielded an OH-PCB 132 metabolite rank order that differed from mouse, rat, and human model systems, with 4′-132 being the major metabolite.

### Comparison of Total PCB 132 Metabolite Levels Across Genotypes and Sex

The analysis of the ΣOH-PCB levels revealed genotype- and sex-dependent differences in the oxidation of PCB 132 by the microsomal preparations under investigation (Figure 3). The ΣOH-PCB levels formed by microsomes from male mice followed the rank order wild-type ∼ *Cyp2f2*-null > *Cyp2a(4/5)bgs*-null. In microsomal preparations from female mice, ΣOH-PCB levels were approximately twofold higher in wild-type mice than in *Cyp2a(4/5)bgs*-null or *Cyp2f2*-null mice. These trends are consistent with a contribution of CYP2F2 and CYP2A(4/5)BGS enzymes to the formation of OH-PCB 132 metabolites. Moreover, like PCB 91 and PCB 95, the ΣOH-PCB levels for PCB 132 were higher in incubations using female mouse microsomes than those using male wild-type or *Cyp2a(4/5)bgs*-null mouse microsomes. This sex difference was statistically significant for microsomal preparations from wild-type mice, with a 2.2-fold difference in the ΣOH-PCB levels. As with PCB 91 and PCB 95, sex differences in PCB 132 metabolism in mice have not been investigated. Our observation suggests that the expression and activity of the P450 isoforms involved in the metabolism of PCB 132 is higher in female mice than male mice in the microsomal preparations investigated.

### Comparison of PCB 132 Metabolite Profiles Across Genotypes and Sex

**Figure 6:**
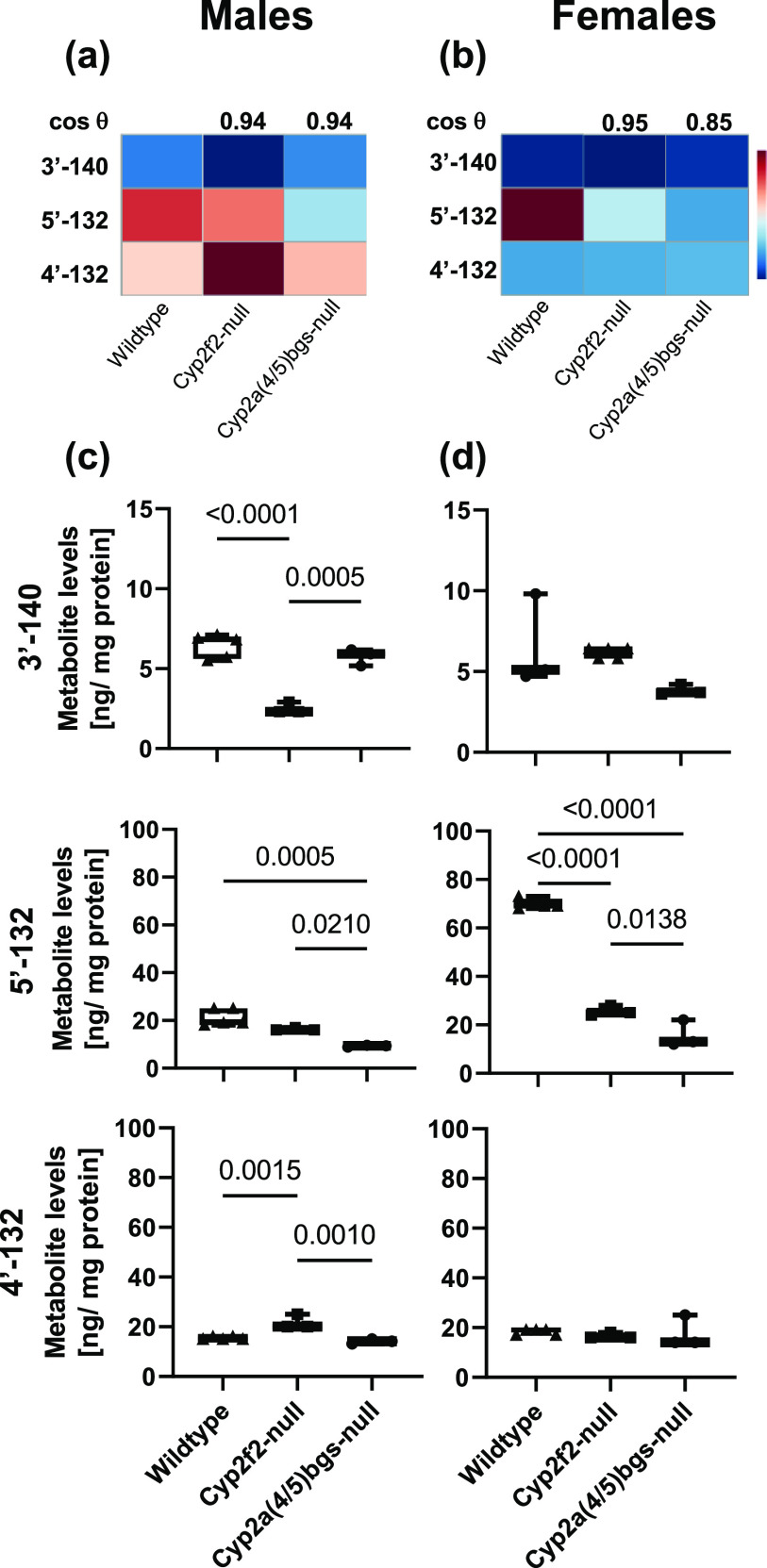
A heatmap-type comparison of the metabolite profiles formed from PCB 132 in incubations with pooled liver microsomes from (A) male and (B) female *Cyp2a(4/5)bgs*-null, *Cyp2f2*-null, and the corresponding wild-type mice reveals differences in the hydroxylated PCB metabolite levels across genotypes. These differences in the metabolite profiles are due to genotype-dependent differences in the levels of individual PCB 132 metabolites in experiments with pooled liver microsomes from (C) male and (D) female mice; significant changes are noted by *p*-values in the figures. The data are expressed as ng/mg protein and, in panels A and B, are visualized using the heatmap function implemented by Metabolanalyst. Metabolite levels were compared using one-way ANOVA. 3′–140, 2,2′,3,4,4′,6′-hexachlorobiphenyl-3-ol; 5′–132, 2,2′,3,3′,4,6′-hexachlorobiphenyl-5′-ol; and 4′–132, 2,2′,3,3′,4,6′-hexachlorobiphenyl-4′-ol.

Similar to PCB 91, and in contrast to PCB 95, OH-PCB 132 profiles formed by microsomal preparations from *Cyp2f2*-null and *Cyp2a(4/5)bgs*-null mice differed from those observed in wild-type mice (Figure 6a and b). The most pronounced differences in the OH-PCB 132 profiles were noted between female *Cyp2a(4/5)bgs*-null and wild-type mouse microsomal incubations, with cos θ = 0.85. In addition, modest differences in the cos θ were observed when comparing the OH-PCB 132 profiles from experiments with microsomes from female *Cyp2f2*-null, male *Cyp2f2*-null, and male *Cyp2a(4/5)bgs*-null mice to the corresponding wild-type mice. As with PCB 91, these results indicate genotype-dependent differences in the formation of individual OH-PCB congeners. Furthermore, the OH-PCB 132 metabolite profiles formed by the different microsomal preparations also showed moderate sex differences, with cos θ ranging from 0.92 for wild-type mouse microsomes to 0.99 for *Cyp2f2*-null mouse microsomes.

### Genotype-Dependent Formation of Individual PCB 132 Metabolites

The 3′-140 levels formed by the microsomal preparations from male mice were significantly lower in *Cyp2f2*-null microsomal preparations than wild-type microsomal preparations (Figure 6c and d). Furthermore, levels of 3′-140 were lower in incubations with *Cyp2f2*-null male mouse microsomes than those with *Cyp2a(4/5)bgs*-null male mouse microsomes. No significant differences in the levels of 3′-140 were observed for microsomes obtained from female mice. The 5′-132 levels formed by the microsomal preparations from male and female mice significantly decreased in the order wild-type > *Cyp2f2*-null > *Cyp2a(4/5)bgs*-null (Figure 6c and d). Moreover, *Cyp2a(4/5)bgs*-null microsomes from male and female mice formed significantly lower levels of 5′-132 than the corresponding *Cyp2f2*-null mouse microsomes. The same general trends across genotypes were observed for 5-91 levels (Figure 4c). The 4′-132 levels formed by the microsomal preparations from male mice were significantly higher in *Cyp2f2*-null mice than either male wild-type or *Cyp2a(4/5)bgs*-null microsomes (Figure 6c and d). Similar genotype-dependent changes in the corresponding *para*-hydroxylated metabolites levels were observed in analogous metabolism studies with PCB 91 and PCB 95 (Figures 4c and 5c). No significant differences in 4′-132 levels were observed for microsomes obtained from female mice. Overall, these results suggest that CYP2F2 is involved in forming 3′-140 in male mice and that CYP2F2 and CYP2A(4/5)BGS enzymes contribute to forming 5′-132 in male and female mice.

### Comparison of PCB 136 Metabolite Levels and Profiles Across Genotypes and Sex

The metabolism of PCB 136 in rat and human liver microsomes and precision-cut liver tissue slices from mice has been studied extensively.^40,41,67,68^ 3-150 (1,2-shift product), 5-136, and 4-136 were typically detected in these *in vitro* model systems. Not all studies report 3-150 because it is a minor metabolite and analytical standards were unavailable, especially for early studies. Like PCB 95, the *para*-hydroxylated metabolite, 4-136, was the major metabolite formed by human CYP2A6.^32^ Rat CYP2B1 and human CYP2B6 preferentially formed 5-136, a *meta*-hydroxylated metabolite.^32,38^ 5-136 and 4-136 were major metabolites in metabolism studies with human liver microsomes.^67,68^ In these earlier studies, the ratio of both metabolites differed depending on the microsomal preparation. 5-136 and 4-136 have also been detected in mice and rats *in vivo* following oral exposure to PCB 136.^18,51,69^ Comparable to the earlier studies, 3-150 (1,2-shift product), 5-136, and 4-136 were formed in incubations of PCB 136 with all microsomal preparations investigated, consistent with established metabolism schemes (Figure 2). The rank order of these metabolites followed the order 5-136 > 4-136 > 3-150 irrespective of the sex or genotype (Table S2). Similar rank orders were observed in studies using other *in vitro* model systems, such as rat liver microsomes^42^ or precision-cut mouse liver tissue slices.^40,41^ In these studies, the extent of the formation of 5-136 increased with the induction of CYP2B enzymes, *e.g.*, with the phenobarbital pretreatment.^40,41^

### Comparison of Total PCB 136 Metabolite Levels and Profiles Across Genotypes and Sex

**Figure 7:**
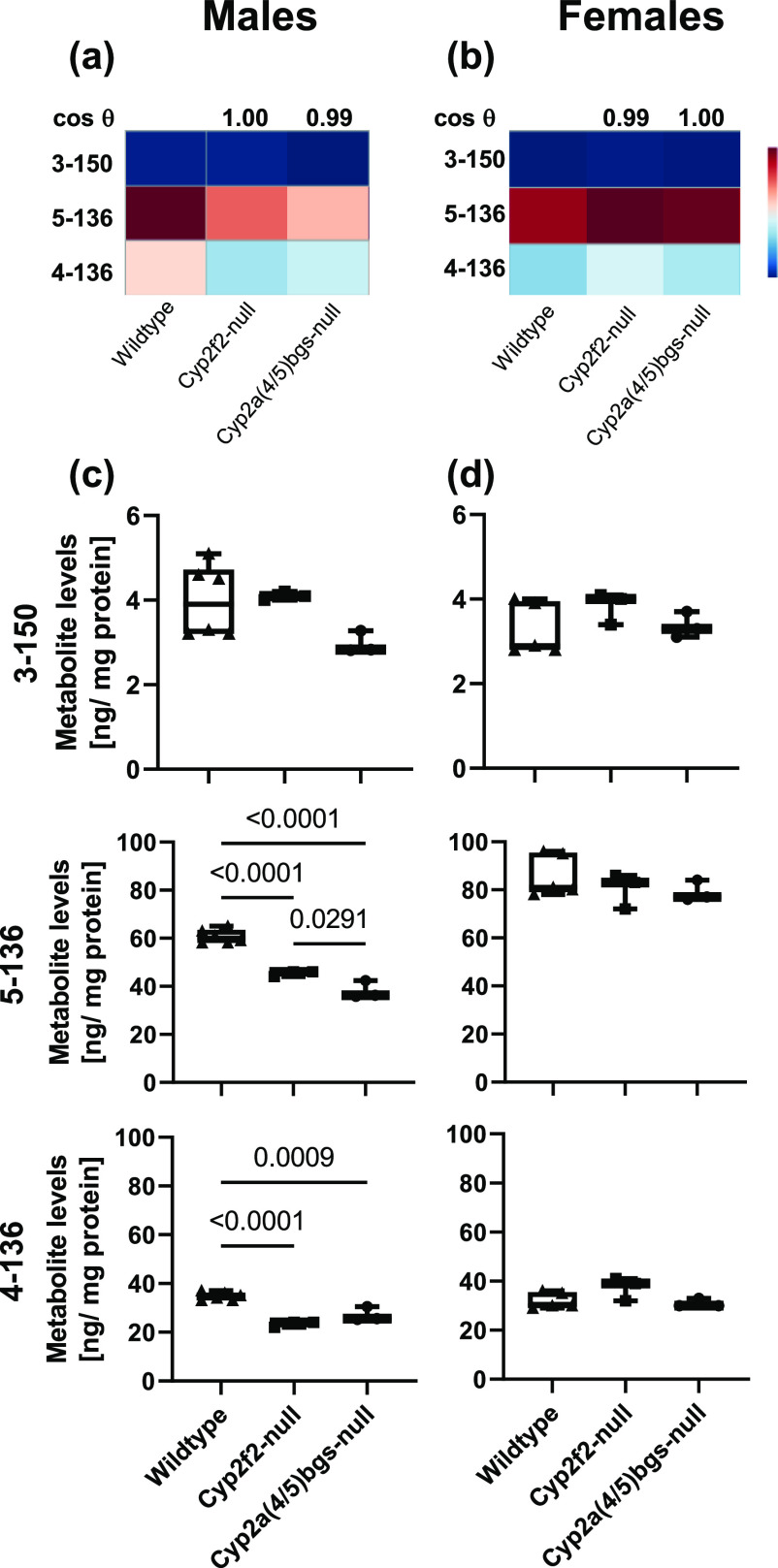
A heatmap-type comparison of the metabolite profiles formed from PCB 136 in incubations with pooled liver microsomes from (A) male and (B) female *Cyp2a(4/5)bgs*-null, *Cyp2f2*-null, and the corresponding wild-type mice reveals differences in the hydroxylated PCB metabolite levels across genotypes. These differences in the metabolite profiles are due to genotype-dependent differences in the levels of individual PCB 136 metabolites in experiments with pooled liver microsomes from (C) male and (D) female mice; significant changes are noted by *p*-values in the figures. The data are expressed as ng/mg protein and, in panels A and B, are visualized using the heatmap function implemented by Metabolanalyst. Metabolite levels were compared using one-way ANOVA. 3–150, 2,2′,3′,4,6,6′-hexachlorobiphenyl-3-ol; 5–136, 2,2′,3,3′,6,6′-hexachlorobiphenyl-5-ol; and 4–136, 2,2′,3,3′,6,6′-hexachlorobiphenyl-4-ol.

As with the other three PCB congeners investigated, the ΣOH-PCB 136 levels were significantly lower in experiments with microsomes from male *Cyp2a(4/5)bgs*-null and *Cyp2f2*-null than those with microsomes from male wild-type mice (Figure 3). Like PCB 91, no statistically significant differences in the ΣOH-PCB 136 levels were observed between microsomal incubations from female mice. This rank order indicates the role of CYP2F2 and CYP2A(4/5)BGS in the oxidation of PCB 136 in the male but not female liver. Despite the significant differences observed for the ΣOH-PCB 136 levels, the OH-PCB 136 profiles were comparable in experiments with microsomes from all genotypes irrespective of sex (Figure 7a and b). As discussed above, the metabolite profiles of PCB 95, which has one *ortho*-chlorine less than PCB 136 and no *para*-chlorine substituent, also did not differ by genotype or sex (cos θ ≥ 0.98, Figure 5a and b). In contrast, the OH-PCB 136 metabolite profiles were sex-dependent in metabolism studies performed with precision-cut liver tissue slices from rats,^41^ possibly due to the sex differences in the hepatic expression of rat CYP2B1.^70^

### Genotype-Dependent Formation of Individual PCB 136 Metabolites

The 3-150 levels formed in microsomal incubations from male and female mice showed no significant genotype effect (Figure 7c and d), unlike the 1,2-shift products formed by the other three PCB congeners investigated (Figures 4–6). In contrast, genotype effects were observed for the other two OH-PCB 136 metabolites. Consistent with the trend observed for the ΣOH-PCB 136 levels (Figure 3), the 5-136 levels formed by the microsomal preparations from male mice significantly decreased in the order wild-type > *Cyp2f2*-null > *Cyp2a(4/5)bgs*-null. The 4-136 levels formed by the microsomal preparations from male wild-type mice were significantly higher than those formed by the microsomal incubations from *Cyp2f2*-null and *Cyp2a(4/5)bgs*-null mice. Studies using female microsomes showed no genotype effect for any metabolite. Overall, the OH-PCB 136 trends in studies with microsomes from male but not female mice indicate a role of CYP2A(4/5)BGS and CYP2F2 in the formation of OH-PCB 136 metabolites.

### Atropselective Metabolism of PCBs by *Cyp2a(4/5)bgs*-null and *Cyp2f2*-null vs Wild-Type Mouse Liver Microsomes

The PCB congeners selected for this study display axial chirality.^27,28^ Chiral PCBs are released into the environment as racemic (1:1) mixtures. They are atropselectively oxidized by mammalian P450 enzymes, resulting in an enrichment of the PCB atropisomer that is metabolized less rapidly *in vivo*. Conversely, the OH-PCB atropisomer formed more rapidly is enriched in these microsomal studies.^27,28^ The atropisomeric enrichment of PCBs is a powerful tool for source apportionment or studying PCB movement through aquatic and terrestrial food webs.^28^ Chiral signatures can also provide insights into differences in the toxicokinetics of chiral PCBs *in vivo*.^71^ They may be useful for assessing the role of specific P450 isoforms in the atropselective metabolism of PCBs. Importantly, PCBs and metabolites atropselectively affect cellular targets, such as the RyR, implicated in PCB toxicity.^29−31,72^

Here, we determined the atropisomeric enrichment of OH-PCBs (as methylated derivatives) from the mouse microsomal incubations to probe genotype-dependent differences in the atropselective metabolism of chiral PCBs. We expect that the knockout of P450 enzymes involved in the formation of specific OH-PCB congeners results in less pronounced atropisomeric enrichment compared to the wild-type. The microsomal metabolism studies were performed with high substrate concentrations to maximize the generation of the OH-PCBs and thus facilitate their atropselective analysis. As a result, the large amount of the parent PCB used in these studies typically masks the atropisomeric enrichment of the parent compound. Therefore, the residues of the parent PCBs were near racemic, as shown in Table 1, and are not discussed further. Although robust EF determinations for the PCB metabolites were not always possible, considerable atropisomeric enrichment was observed for most OH-PCB metabolites, as described below for the individual PCB congeners.

**Table 1:** Enantiomeric Fraction (EF) Values of Hydroxylated PCB Metabolites Formed from Chiral PCBs Incubated with Mouse Liver Microsomes from Male and Female *Cyp2a(4/5)bgs*-null, *Cyp2f2*-null, and Wild-Type (WT) Mice^a^

	male mice					female mice				
mouse model	parent PCB	1,2-shift product	5-OH-PCB	4′-OH-PCB 95	4-OH-PCB	parent PCB	1,2-shift product	5-OH-PCB	4′-OH-PCB 95	4-OH-PCB
PCB 91^b^										
*Cyp2a*(4/5)*bgs*-null	0.49 ± 0.01	(0.96 ± 0.01)	0.50 ± 0.03		0.84 ± 0.01	0.49 ± 0.01		0.56 ± 0.01		0.85 ± 0.01
WT	0.49 ± 0.01	(0.95 ± 0.02)	0.45 ± 0.01		0.83 ± 0.03	0.49 ± 0.01	0.94 ± 0.01	0.34 ± 0.03		0.79 ± 0.04
PCB 95^b^										
*Cyp2a*(4/5)*bgs*-null	0.50 ± 0.01			0.66 ± 0.04		0.50 ± 0.01			0.65 ± 0.03	
WT	0.50 ± 0.01			0.65 ± 0.10		0.50 ± 0.01			0.65 ± 0.05	
PCB 132										
*Cyp2a*(4/5)*bgs*-null	0.50 ± 0.01	0.37 ± 0.02	0.49 ± 0.03			0.49 ± 0.01		0.66 ± 0.01		
*Cyp2f2*-null							0.38 ± 0.02			
WT	0.50 ± 0.01		0.31 ± 0.05			0.52 ± 0.02		0.28 ± 0.09		
PCB 136										
*Cyp2a*(4/5)*bgs*-null			0.70 ± 0.02		0.78 ± 0.01			0.72 ± 0.03		0.71 ± 0.01
*Cyp2f2*-null			0.56 ± 0.02		0.54 ± 0.01			0.62 ± 0.01		0.65 ± 0.03
WT			0.70 ± 0.03		0.72 ± 0.04			0.70 ± 0.04		0.65 ± 0.08

a See Figures S1–S6 for representative chromatograms. Data are expressed as the mean ± SD, *n* = 3. Metabolism studies with mouse liver microsomal preparations from *Cyp2a(4/5)bgs*-null, *Cyp2f2*-null, and the corresponding WT mice were performed using the following incubation conditions: 50 μM PCB, 30 min of incubation at 37 °C, 0.1 mg/mL microsomal protein content, and 1 mM NADPH. Microsomes were prepared from wild-type C57BL/6 mice and knockout mice with the same genetic background. Atropselective analyses were performed as described in the Experimental Procedures. EF values were calculated by the drop valley method as EF = area E_1_/(area E_1_ + area E_2_), where area E_1_ and area E_2_ denote the peak areas of the first- (E_1_) and second-eluting (E_2_) atropisomers, respectively.^59^ EF values in brackets indicate poor peak resolution, increasing the uncertainty of the EF value determination. Empty fields indicate that the EF values of the metabolites could not be determined.

b EF values for metabolites formed in incubations with microsomes from *Cyp2f2*-null mice could not be determined.

### Atropisomeric Enrichment of OH-PCB 91 Metabolites in *Cyp2a(4/5)bgs*-null vs Wild-Type Mouse Liver Microsomes

The 1,2-shift metabolite of PCB 91, namely, 3-100, showed considerable enrichment of E_1_-3-100 in microsomal incubations with microsomes from male and female wild-type mice and male *Cyp2a(4/5)bgs*-null mice. No genotype or sex effects were observed. Similarly, E_1_-3-100 was enriched in metabolism studies with human CYP2A6 and rat liver microsomes.^32,58^ The atropisomeric enrichment of this PCB 91 metabolite has not been characterized in mice or other animal models *in vivo*.^62^ Consistent with our interpretation of the 3-100 levels above, the chiral signatures indicate that CYP2A(4/5)BGS enzymes are involved in the atropselective formation of 3-100 in the microsomal preparations investigated.

E_1_-4-91 was enriched in microsomal incubations from male and female wild-type and *Cyp2a(4/5)bgs*-null mice, and no genotype or sex effect was noted (Table 1). This observation suggests that CYP2A(4/5)BGS enzymes do not contribute to the atropselective formation of 4-91. Consistent with the present study, enrichment of E_1_-4-91 was also observed in the blood, liver, and feces of mice exposed orally to racemic PCB 91.^62^ Moreover, studies with recombinant human P450 enzymes reveal P450 isoform-specific metabolism, with E_1_-4-91 being enriched in experiments with recombinant CYP2B6 and CYP2E1 and E_2_-4-91 being enriched in studies with CYP2A6.^32^

E_2_-5-91 was enriched in metabolism studies with microsomes obtained from wild-type mice (Table 1). Interestingly, more pronounced atropisomeric enrichment of E_2_-5-91 was observed in experiments with microsomes from female wild-type mice than those with microsomes from male wild-type mice. In contrast, 5-91 was racemic in incubations with microsomes from male *Cyp2a(4/5)bgs*-null mice, and E_1_-5-91 was enriched in studies with microsomes from female *Cyp2a(4/5)bgs*-null mice. These differences in the extent and direction of the atropisomeric enrichment indicate the role of CYP2A(4/5)BGS enzymes in the formation of E_2_-5-91, consistent with our interpretation of the genotype differences of the 5-91 levels (Figure 4).

### Atropisomeric Enrichment of OH-PCB 95 Metabolites in *Cyp2a(4/5)bgs*-null vs Wild-Type Mouse Liver Microsomes

We were able to determine the EF values of 4′-95 in incubations with microsomes from wild-type and *Cyp2a(4/5)bgs*-null mice. E_1_-4′-95 was enriched in these microsomal preparations, and no genotype or sex effect was noted. Thus, the CYP2A(4/5)BGS enzymes do not appear to contribute to the formation of 4′-95. Although the formation of this metabolite has been documented in mice and other organisms,^18,63,65,66,73^ its atropisomeric enrichment in mice has not been reported to date. However, studies with recombinant P450 enzymes revealed the preferential formation of E_2_-4′-95 and E_1_-4′-95 by human CYP2A6 and CYP2E1, respectively.^32^

### Atropisomeric Enrichment of OH-PCB 132 Metabolites in *Cyp2a(4/5)bgs*-null vs Wild-Type Mouse Liver Microsomes

We assessed the atropisomeric enrichment of 5′-132 in incubations using *Cyp2a(4/5)bgs*-null and wild-type liver microsomes (Table 1). The overall trends in the extent and direction of the atropisomeric enrichment of this metabolite were similar to those discussed above for PCB 91. Briefly, E_2_-5′-132 was enriched in incubations with wild-type microsomes, with comparable EF values for microsomal preparations from male and female mice. Metabolism studies with microsomes from wild-type mice, precision-cut mouse liver tissue slices from phenobarbital-pretreated mice, rat CYP2B1, or rat liver microsomes also showed enrichment of E_2_-5′-132.^38,41,58^

A different direction of the atropisomeric enrichment of 5′-132 was observed in studies using microsomes from *Cyp2a(4/5)bgs*-null mice. In addition, the chiral signature of 5′-132 was near racemic in experiments with microsomes from male mice, whereas E_1_-5′-132 was enriched in studies using microsomes from female mice. These differences between wild-type and *Cyp2a(4/5)bgs*-null mouse microsomal incubations and studies using precision-cut mouse liver tissue slices from phenobarbital pretreated mice^40^ indicate the role of CYP2A(4/5)BGS enzymes in the formation of E_2_-5′-132, consistent with our interpretation of the PCB 132 metabolite levels (Figure 6).

### Atropisomeric Enrichment of OH-PCB 136 Metabolites in *Cyp2a(4/5)bgs*-null and *Cyp2f2*-null vs Wild-Type Mouse Liver Microsomes

Incubations with male and female microsomes from all three mouse models preferentially formed E_1_-5-136 and E_1_-4-136 (Table 1), consistent with earlier *in vitro* metabolism studies using mouse microsomes or precision-cut mouse liver tissue slices.^40,68^ It is noteworthy that a different direction of the atropisomeric enrichment was observed in metabolism and disposition studies with other species, with E_2_-5-136 and E_2_-4-136 being enriched.^27^

The extent of the atropisomeric enrichment of E_1_-5-136 showed no genotype or sex effect in experiments using microsomes from wild-type and *Cyp2a(4/5)bgs*-null mice (Table 1). In contrast, less pronounced enrichment of E_1_-5-136 was observed in *Cyp2f2*-null microsomal preparations, with EF values being lower in incubations using male microsomes than those using female microsomes. These differences support the role of CYP2F2 in the atropselective formation of 5-136, consistent with our interpretation of the differences between the 5–136 levels discussed above (Figure 7). However, these results do not support the role of CYP2A(4/5)BGS enzymes in the atropselective formation of these PCB 136 metabolites, in contrast to our interpretation of the 5-136 levels.

The EF values of 4-136 also showed genotype and sex differences (Table 1). The enrichment of E_1_-4-136 was more pronounced in incubations using *Cyp2a(4/5)bgs*-null than those using wild-type mouse microsomes, irrespective of sex, which does not suggest that CYP2A(4/5)BGS has a role in the formation of 4-136. Interestingly, the extent of the atropisomeric enrichment of E_1_-4-136 was less pronounced in incubations using male *Cyp2f2*-null microsomes than those using *Cyp2a(4/5)bgs*-null and wild-type microsomes. Similarly, lower 4-136 levels were observed in male *Cyp2f2*-null preparations compared to *Cyp2a(4/5)bgs*-null and wild-type preparations (Figure 3). Together, these results are consistent with a role of CYP2F2 in the atropselective formation of 4-136 in male but not female mice.

### The Role of CYP2A(4/5)BGS and CYP2F2 Enzymes in the Metabolism of Chiral PCBs: General Considerations

Based on our analysis of the total OH-PCB levels, the OH-PCB profiles, the levels of individual OH-PCB congeners, and, where available, the chiral OH-PCB signatures, all four PCB congeners appear to be atropselectively metabolized by CYP2F2 and CYP2A(4/5)BGS enzymes. For example, 3-100, 5-91, 3-103, 3′-140, 5′-132, 5-136, and 4-136 are formed at lower levels in microsomal preparations from male or female *Cyp2f2*-null mouse microsomes. Similarly, 5-91, 3-103, 4′-95, 4-95, 5′-132, 5-136, and 4-136 are formed at lower levels in microsomal preparations from male or female *Cyp2a(4/5)bgs*-null mouse microsomes. These differences in OH-PCB levels are consistent with a role of the respective P450 enzymes in the formation of the metabolite. However, we also observed higher levels of certain OH-PCB congeners in microsomal preparations from *Cyp2f2*-null and *Cyp2a(4/5)bgs*-null mice than wild-type microsomes. Compensatory changes in the liver due to the knockout of *Cyp2f2* or *Cyp2a(4/5)bgs* are a likely explanation for this observation. These compensatory changes may result in an increased expression and activity of other P450 isoforms involved in the formation of specific OH-PCB congeners, thus increasing their formation in the microsomal studies despite the deletion of *Cyp2f2* or *Cyp2a(4/5)bgs*. Previous immunoblotting studies showed an absence of compensatory changes in several P450 proteins, including CYP1A, CYP2E1, and CYP3A in *Cyp2f2*-null mice^54^ and CYP1A, CYP2C, CYP2E1, CYP3A, and P450 reductase in *Cyp2a(4/5)bgs*-null mice.^55^ To further explore this possibility, a more in-depth characterization of the P450 enzyme profiles is needed for both mouse models. Another consideration is that the overall decrease in the rates of PCB metabolism in the null mice would leave more substrates available for metabolism by the remaining P450 enzymes, resulting in a higher rate of formation of the specific metabolites formed by the latter enzymes.

We also observed that PCB metabolism by microsomal preparations from *Cyp2f2*-null and *Cyp2a(4/5)bgs*-null mice is congener-specific, resulting in OH-PCB profiles that are different between null and wild-type mice for PCB 91 and PCB 132, as indicated by the similarity coefficient. In contrast, the metabolite profiles of PCB 95 and PCB 136, PCB congeners without a *para*-chlorine group, were comparable between microsomes from wild-type, *Cyp2f2*-null, and *Cyp2a(4/5)bgs*-null mice. These PCB-structure-dependent differences in the OH-PCB profiles may result from subtle differences in the interactions of the substrate (or intermediates, such as PCB arenes) with the active site of the P450 enzymes and are caused by the presence or absence of the *para*-chlorine substituent. Therefore, additional studies are needed to characterize specific P450 isoforms that contribute to the oxidation of chiral PCBs in the mouse liver.

Overall, our results revealed that the atropselective metabolism of chiral PCBs in mice is complex and likely involves several P450 isoforms, including CYP2F2 and/or CYP2A(4/5)BGS. More importantly, our results also demonstrate that both *Cyp2f2*-null and *Cyp2a(4/5)bgs*-null mouse models can be used to study how a loss of metabolic function affects the neurotoxicity of PCB congeners with multiple *ortho*-chlorine substituents across the lifetime. Specifically, *Cyp2a(4/5)bgs*-null mice may be a useful model to study neurotoxic outcomes following oral PCB exposure, whereas *Cyp2f2*-null mice are of interest to examine how CYP2F2-mediated metabolism in the lung affects PCB neurotoxicity following inhalation exposure. Because of the growing recognition that inhalation is an important and current route of PCB exposure in humans, *Cyp2f2*-null mice may be an important model to study how metabolism in the lung affects the disposition and neurotoxicity of inhaled PCBs.

## Supporting Information Available

Assessment of the intraday and interday variation of metabolism studies, levels and similarity coefficients of hydroxylated PCB metabolites formed in the microsome incubations, description of chiral capillary gas chromatography columns, quality assurance and quality control information for the chemical analyses, and representative chromatograms from the atropselective analyses (PDF)

## References

[ref1] Vorkamp K. An overlooked environmental issue? A review of the inadvertent formation of PCB-11 and other PCB congeners and their occurrence in consumer products and in the environment. Sci. Total Environ. 2016, 541, 1463–1476. 10.1016/j.scitotenv.2015.10.019. 26490526

[ref2] Grimm F. A.; Hu D.; Kania-Korwel I.; Lehmler H. J.; Ludewig G.; Hornbuckle K. C.; Duffel M. W.; Bergman A.; Robertson L. W. Metabolism and metabolites of polychlorinated biphenyls. Crit. Rev. Toxicol. 2015, 45 (3), 245–272. 10.3109/10408444.2014.999365. 25629923 PMC4383295

[ref3] Schettgen T.; Esser A.; Alt A.; Randerath I.; Kraus T.; Ziegler P. Decomposition products of the initiator bis(2,4-dichlorobenzoyl)peroxide in the silicone industry: Human biomonitoring in plasma and urine of workers. Environ. Sci. Technol. 2022, 56, 8518–8527. 10.1021/acs.est.2c01530. 35671459

[ref4] Herrick R. F.; Stewart J. H.; Allen J. G. Review of PCBs in US schools: a brief history, an estimate of the number of impacted schools, and an approach for evaluating indoor air samples. Environ. Sci. Pollut. Res. Int. 2016, 23 (3), 1975–1985. 10.1007/s11356-015-4574-8. 25940477 PMC4635108

[ref5] Herrick R. F.; Meeker J. D.; Altshul L. Serum PCB levels and congener profiles among teachers in PCB-containing schools: A pilot study. Environ. Health 2011, 10, 56 10.1186/1476-069X-10-56. 21668970 PMC3136408

[ref6] Marek R. F.; Thorne P. S.; Herkert N. J.; Awad A. M.; Hornbuckle K. C. Airborne PCBs and OH-PCBs inside and outside urban and rural U.S. schools. Environ. Sci. Technol. 2017, 51 (14), 7853–7860. 10.1021/acs.est.7b01910. 28656752 PMC5777175

[ref7] Schecter A.; Colacino J.; Haffner D.; Patel K.; Opel M.; Papke O.; Birnbaum L. Perfluorinated compounds, polychlorinated biphenyls, and organochlorine pesticide contamination in composite food samples from Dallas, Texas, USA. Environ. Health Perspect. 2010, 118 (6), 796–802. 10.1289/ehp.0901347. 20146964 PMC2898856

[ref8] Chen X.; Lin Y.; Dang K.; Puschner B. Quantification of polychlorinated biphenyls and polybrominated diphenyl ethers in commercial cows’ milk from California by gas chromatography-triple quadruple mass spectrometry. PLoS One 2017, 12 (1), e0170129 10.1371/journal.pone.0170129. 28085917 PMC5234792

[ref9] Shin E. S.; Nguyen K. H.; Kim J.; Kim C. I.; Chang Y. S. Progressive risk assessment of polychlorinated biphenyls through a Total Diet Study in the Korean population. Environ. Pollut. 2015, 207, 403–412. 10.1016/j.envpol.2015.08.051. 26470055

[ref10] Sethi S.; Morgan R. K.; Feng W.; Lin Y. P.; Li X. S.; Luna C.; Koch M.; Bansal R.; Duffel M. W.; Puschner B.; Zoeller R. T.; Lehmler H. J.; Pessah I. N.; Lein P. J. Comparative analyses of the 12 most abundant PCB congeners detected in human maternal serum for activity at the thyroid hormone receptor and ryanodine receptor. Environ. Sci. Technol. 2019, 53 (7), 3948–3958. 10.1021/acs.est.9b00535. 30821444 PMC6457253

[ref11] Mitchell M. M.; Woods R.; Chi L. H.; Schmidt R. J.; Pessah I. N.; Kostyniak P. J.; LaSalle J. M. Levels of select PCB and PBDE congeners in human postmortem brain reveal possible environmental involvement in 15q11-q13 duplication autism spectrum disorder. Environ. Mol. Mutagen. 2012, 53 (8), 589–598. 10.1002/em.21722. 22930557 PMC3739306

[ref12] Dewailly E.; Mulvad G.; Pedersen H. S.; Ayotte P.; Demers A.; Weber J. P.; Hansen J. C. Concentration of organochlorines in human brain, liver, and adipose tissue autopsy samples from Greenland. Environ. Health Perspect. 1999, 107 (10), 823–828. 10.1289/ehp.99107823. PMC1566611 10504150

[ref13] Li X.; Hefti M. M.; Marek R. F.; Hornbuckle K. C.; Wang K.; Lehmler H. J. Assessment of polychlorinated biphenyls and their hydroxylated metabolites in postmortem human brain samples: age and brain region differences. Environ. Sci. Technol. 2022, 56 (13), 9515–9526. 10.1021/acs.est.2c00581. 35658127 PMC9260965

[ref14] Anezaki K.; Nakano T. Concentration levels and congener profiles of polychlorinated biphenyls, pentachlorobenzene, and hexachlorobenzene in commercial pigments. Environ. Sci. Poll. Res. Int. 2014, 21 (2), 998–1009. 10.1007/s11356-013-1977-2. 23852585

[ref15] Saktrakulkla P.; Lan T.; Hua J.; Marek R. F.; Thorne P. S.; Hornbuckle K. C. Polychlorinated biphenyls in food. Environ. Sci. Technol. 2020, 54 (18), 11443–11452. 10.1021/acs.est.0c03632. 32816464 PMC7759298

[ref16] Ampleman M. D.; Martinez A.; DeWall J.; Rawn D. F.; Hornbuckle K. C.; Thorne P. S. Inhalation and dietary exposure to PCBs in urban and rural cohorts via congener-specific measurements. Environ. Sci. Technol. 2015, 49 (2), 1156–1164. 10.1021/es5048039. 25510359 PMC4303332

[ref17] Grimm F. A.; He X.; Teesch L. M.; Lehmler H. J.; Robertson L. W.; Duffel M. W. Tissue distribution, metabolism, and excretion of 3,3′-dichloro-4′-sulfooxy-biphenyl in the rat. Environ. Sci. Technol. 2015, 49 (13), 8087–8095. 10.1021/acs.est.5b01499. 26046945 PMC4496304

[ref18] Kania-Korwel I.; Lukasiewicz T.; Barnhart C. D.; Stamou M.; Chung H.; Kelly K. M.; Bandiera S.; Lein P. J.; Lehmler H.-J. Congener-specific disposition of chiral polychlorinated biphenyls in lactating mice and their offspring: Implications for PCB developmental neurotoxicity. Toxicol. Sci. 2017, 158 (1), 101–115. 10.1093/toxsci/kfx071. 28431184 PMC6070089

[ref19] Pessah I. N.; Lein P. J.; Seegal R. F.; Sagiv S. K. Neurotoxicity of polychlorinated biphenyls and related organohalogens. Acta Neuropathol. 2019, 138 (3), 363–387. 10.1007/s00401-019-01978-1. 30976975 PMC6708608

[ref20] Klocke C.; Lein P. J. Evidence implicating non-dioxin-like congeners as the key mediators of polychlorinated biphenyl (PCB) developmental neurotoxicity. Int. J. Mol. Sci. 2020, 21 (3), 1013 10.3390/ijms21031013. 32033061 PMC7037228

[ref21] Pessah I. N.; Cherednichenko G.; Lein P. J. Minding the calcium store: Ryanodine receptor activation as a convergent mechanism of PCB toxicity. Pharmacol. Ther. 2010, 125 (2), 260–285. 10.1016/j.pharmthera.2009.10.009. 19931307 PMC2823855

[ref22] Niknam Y.; Feng W.; Cherednichenko G.; Dong Y.; Joshi S. N.; Vyas S. M.; Lehmler H.-J.; Pessah I. N. Structure-activity relationship of select *meta*- and *para*-hydroxylated non-dioxin-like polychlorinated biphenyls: from single RyR1 channels to muscle dysfunction. Toxicol. Sci. 2013, 136 (2), 500–513. 10.1093/toxsci/kft202. 24014653 PMC3858193

[ref23] Holland E. B.; Feng W.; Zheng J.; Dong Y.; Li X.; Lehmler H.-J.; Pessah I. N. An extended structure-activity relationship of non-dioxin-like PCBs evaluates and supports modeling predictions and identifies picomolar potency of PCB 202 towards ryanodine receptors. Toxicol. Sci. 2017, 155 (1), 170–181. 10.1093/toxsci/kfw189. 27655348 PMC5216651

[ref24] Pessah I. N.; Hansen L. G.; Albertson T. E.; Garner C. E.; Ta T. A.; Do Z.; Kim K. H.; Wong P. W. Structure-activity relationship for noncoplanar polychlorinated biphenyl congeners toward the ryanodine receptor-Ca^2+^ channel complex type 1 (RyR1). Chem. Res. Toxicol. 2006, 19 (1), 92–101. 10.1021/tx050196m. 16411661

[ref25] Meerts I. A. T. M.; Hoving S.; van den Berg J. H. J.; Weijers B. M.; Swarts H. J.; van der Beek E. M.; Bergman A.; Koeman J. H.; Brouwer A. Effects of in utero exposure to 4-hydroxy-2,3,3′,4′,5-pentachlorobiphenyl (4-OH-CB107) on developmental landmarks, steroid hormone levels, and female estrous cyclicity in rats. Toxicol. Sci. 2004, 82 (1), 259–267. 10.1093/toxsci/kfh251. 15310862

[ref26] Meerts I. A. T. M.; Lilienthal H.; Hoving S.; van den Berg J. H. J.; Weijers B. M.; Bergman A.; Koeman J. H.; Brouwer A. Developmental exposure to 4-hydroxy-2,3,3′,4′,5-pentachlorobiphenyl (4-OH-CB107): Long-term effects on brain development, behavior, and brain stem auditory evoked potentials in rats. Toxicol. Sci. 2004, 82 (1), 207–218. 10.1093/toxsci/kfh252. 15310863

[ref27] Kania-Korwel I.; Lehmler H. J. Chiral polychlorinated biphenyls: absorption, metabolism and excretion-a review. Environ. Sci. Pollut. Res. Int. 2016, 23 (3), 2042–2057. 10.1007/s11356-015-4150-2. 25651810 PMC4527964

[ref28] Lehmler H.-J.; Harrad S. J.; Huhnerfuss H.; Kania-Korwel I.; Lee C. M.; Lu Z.; Wong C. S. Chiral polychlorinated biphenyl transport, metabolism, and distribution: A review. Environ. Sci. Technol. 2010, 44 (8), 2757–2766. 10.1021/es902208u. 20384371 PMC2855137

[ref29] Pessah I. N.; Lehmler H.-J.; Robertson L. W.; Perez C. F.; Cabrales E.; Bose D. D.; Feng W. Enantiomeric specificity of (−)-2,2′,3,3′,6,6′-hexachlorobiphenyl toward ryanodine receptor types 1 and 2. Chem. Res. Toxicol. 2009, 22, 201–207. 10.1021/tx800328u. 18954145 PMC2662366

[ref30] Yang D.; Kania-Korwel I.; Ghogha A.; Chen H.; Stamou M.; Bose D. D.; Pessah I. N.; Lehmler H. J.; Lein P. J. PCB 136 atropselectively alters morphometric and functional parameters of neuronal connectivity in cultured rat hippocampal neurons via ryanodine receptor-dependent mechanisms. Toxicol. Sci. 2014, 138 (2), 379–392. 10.1093/toxsci/kft334. 24385416 PMC4007107

[ref31] Feng W.; Zheng J.; Robin G.; Dong Y.; Ichikawa M.; Inoue Y.; Mori T.; Nakano T.; Pessah I. N. Enantioselectivity of 2,2′,3,5′,6-pentachlorobiphenyl (PCB 95) atropisomers toward ryanodine receptors (RyRs) and their influences on hippocampal neuronal networks. Environ. Sci. Technol. 2017, 51 (24), 14406–14416. 10.1021/acs.est.7b04446. 29131945 PMC6251309

[ref32] Uwimana E.; Ruiz P.; Li X.; Lehmler H. J. Human CYP2A6, CYP2B6, and CYP2E1 atropselectively metabolize polychlorinated biphenyls to hydroxylated metabolites. Environ. Sci. Technol. 2019, 53 (4), 2114–2123. 10.1021/acs.est.8b05250. 30576102 PMC6380921

[ref33] McGraw J. E.; Waller D. P. Specific human CYP 450 isoform metabolism of a pentachlorobiphenyl (PCB-IUPAC# 101). Biochem. Biophys. Res. Commun. 2006, 344 (1), 129–133. 10.1016/j.bbrc.2006.03.122. 16616008

[ref34] Shimada T.; Kakimoto K.; Takenaka S.; Koga N.; Uehara S.; Murayama N.; Yamazaki H.; Kim D.; Guengerich F. P.; Komori M. Roles of human CYP2A6 and monkey CYP2A24 and 2A26 cytochrome P450 enzymes in the oxidation of 2,5,2′,5′-tetrachlorobiphenyl. Drug Metab. Dispos. 2016, 44 (12), 1899–1909. 10.1124/dmd.116.072991. 27625140 PMC6047209

[ref35] Warner N. A.; Martin J. W.; Wong C. S. Chiral polychlorinated biphenyls are biotransformed enantioselectively by mammalian cytochrome P-450 isozymes to form hydroxylated metabolites. Environ. Sci. Technol. 2009, 43 (1), 114–121. 10.1021/es802237u. 19209593

[ref36] Ariyoshi N.; Oguri K.; Koga N.; Yoshimura H.; Funae Y. Metabolism of highly persistent PCB congener, 2,4,5,2′,4′,5′-hexachlorobiphenyl, by human CYP2B6. Biochem. Biophys. Res. Commun. 1995, 212 (2), 455–460. 10.1006/bbrc.1995.1991. 7626059

[ref37] Ohta C.; Haraguchi K.; Kato Y.; Koga N. In vitro metabolism of 2,2’,3,4’,5,5′,6-heptachlorobiphenyl (CB187) by liver microsomes from rats, hamsters and guinea pigs. Xenobiotica 2005, 35 (4), 319–330. 10.1080/00498250500087507. 16019954

[ref38] Lu Z.; Kania-Korwel I.; Lehmler H. J.; Wong C. S. Stereoselective formation of mono- and dihydroxylated polychlorinated biphenyls by rat cytochrome P450 2B1. Environ. Sci. Technol. 2013, 47 (21), 12184–92. 10.1021/es402838f. 24060104 PMC3870094

[ref39] Lu Z.; Wong C. S. Factors affecting phase I stereoselective biotransformation of chiral polychlorinated biphenyls by rat cytochrome P-450 2B1 isozyme. Environ. Sci. Technol. 2011, 45 (19), 8298–8305. 10.1021/es200673q. 21863805

[ref40] Wu X.; Duffel M.; Lehmler H.-J. Oxidation of polychlorinated biphenyls by liver tissue slices from phenobarbital-pretreated mice is congener-specific and atropselective. Chem. Res. Toxicol. 2013, 26 (11), 1642–1651. 10.1021/tx400229e. 24107130 PMC3857157

[ref41] Wu X.; Kania-Korwel I.; Chen H.; Stamou M.; Dammanahalli K. J.; Duffel M.; Lein P. J.; Lehmler H.-J. Metabolism of 2,2′,3,3′,6,6′-hexachlorobiphenyl (PCB 136) atropisomers in tissue slices from phenobarbital or dexamethasone-induced rats is sex-dependent. Xenobiotica 2013, 43 (11), 933–947. 10.3109/00498254.2013.785626. 23581876 PMC3878182

[ref42] Wu X.; Pramanik A.; Duffel M. W.; Hrycay E. G.; Bandiera S. M.; Lehmler H.-J.; Kania-Korwel I. 2,2′,3,3′,6,6′-Hexachlorobiphenyl (PCB 136) is enantioselectively oxidized to hydroxylated metabolites by rat liver microsomes. Chem. Res. Toxicol. 2011, 24 (12), 2249–2257. 10.1021/tx200360m. 22026639 PMC3243785

[ref43] Waller S. C.; He Y. A.; Harlow G. R.; He Y. Q.; Mash E. A.; Halpert J. R. 2,2′,3,3′,6,6′-Hexachlorobiphenyl hydroxylation by active site mutants of cytochrome P450 2B1 and 2B11. Chem. Res. Toxicol. 1999, 12, 690–699. 10.1021/tx990030j. 10458702

[ref44] Kaminsky L. S.; Kennedy M. W.; Adams S. M.; Guengerich F. P. Metabolism of dichlorobiphenyls by highly purified isozymes of rat liver cytochrome P-450. Biochemistry 1981, 20, 7379–7384. 10.1021/bi00529a009. 6798990

[ref45] Uwimana E.; Cagle B.; Yeung C.; Li X.; Patterson E. V.; Doorn J. A.; Lehmler H. J. Atropselective oxidation of 2,2′,3,3′,4,6′-hexachlorobiphenyl (PCB 132) to hydroxylated metabolites by human liver microsomes and its implications for PCB 132 neurotoxicity. Toxicol. Sci. 2019, 171 (2), 406–420. 10.1093/toxsci/kfz150. 31268529 PMC6760323

[ref46] Uwimana E.; Li X.; Lehmler H.-J. Human liver microsomes atropselectively metabolize 2,2′,3,4′,6-pentachlorobiphenyl (PCB 91) to a 1,2-shift product as the major metabolite. Environ. Sci. Technol. 2018, 52 (10), 6000–6008. 10.1021/acs.est.8b00612. 29659268 PMC5966832

[ref47] Uwimana E.; Li X.; Lehmler H.-J. 2,2′,3,5′,6-Pentachlorobiphenyl (PCB 95) is atropselectively metabolized to para-hydroxylated metabolites by human liver microsomes. Chem. Res. Toxicol. 2016, 29 (12), 2108–2110. 10.1021/acs.chemrestox.6b00371. 27989147 PMC5175585

[ref48] Klinefelter K.; Hooven M. K.; Bates C.; Colter B. T.; Dailey A.; Infante S. K.; Kania-Korwel I.; Lehmler H. J.; Lopez-Juarez A.; Ludwig C. P.; Curran C. P. Genetic differences in the aryl hydrocarbon receptor and CYP1A2 affect sensitivity to developmental polychlorinated biphenyl exposure in mice: relevance to studies of human neurological disorders. Mamm. Genome 2018, 29 (1–2), 112–127. 10.1007/s00335-017-9728-1. 29197979 PMC6425730

[ref49] Curran C. P.; Vorhees C. V.; Williams M. T.; Genter M. B.; Miller M. L.; Nebert D. W. In utero and lactational exposure to a complex mixture of polychlorinated biphenyls: toxicity in pups dependent on the Cyp1a2 and Ahr genotypes. Toxicol. Sci. 2011, 119 (1), 189–208. 10.1093/toxsci/kfq314. 20961953 PMC3003831

[ref50] Curran C. P.; Nebert D. W.; Genter M. B.; Patel K. V.; Schaefer T. L.; Skelton M. R.; Williams M. T.; Vorhees C. V. In utero and lactational exposure to PCBs in mice: adult offspring show altered learning and memory depending on Cyp1a2 and Ahr genotypes. Environ. Health Perspect. 2011, 119 (9), 1286–1293. 10.1289/ehp.1002965. 21571617 PMC3230394

[ref51] Wu X.; Barnhart C.; Lein P. J.; Lehmler H. J. Hepatic metabolism affects the atropselective disposition of 2,2′,3,3′,6,6′-hexachlorobiphenyl (PCB 136) in mice. Environ. Sci. Technol. 2015, 49 (1), 616–625. 10.1021/es504766p. 25420130 PMC4291784

[ref52] Li X.; Zhang C.; Wang K.; Lehmler H.-J. Fatty liver and impaired hepatic metabolism alter the congener-specific distribution of polychlorinated biphenyls (PCBs) in mice with a liver-specific deletion of cytochrome P450 reductase. Environ. Pollut. 2020, 266, 115233 10.1016/j.envpol.2020.115233. 32712482 PMC7492420

[ref53] Egusquiza R. J.; Ambrosio M. E.; Wang S. G.; Kay K. M.; Zhang C.; Lehmler H. J.; Blumberg B. Evaluating the role of the steroid and xenobiotic receptor (SXR/PXR) in PCB-153 metabolism and protection against associated adverse effects during perinatal and chronic exposure in mice. Environ. Health Perspect. 2020, 128 (4), 047011 10.1289/EHP6262. 32352317 PMC7228131

[ref54] Li L.; Wei Y.; Van Winkle L.; Zhang Q.-Y.; Zhou X.; Hu J.; Xie F.; Kluetzman K.; Ding X. Generation and characterization of a *Cyp2f2*-null mouse and studies on the role of CYP2F2 in naphthalene-induced toxicity in the lung and nasal olfactory mucosa. J. Pharmacol. Exp. Ther. 2011, 339 (1), 62 10.1124/jpet.111.184671. 21730012 PMC3186285

[ref55] Wei Y.; Li L.; Zhou X.; Zhang Q.-Y.; Dunbar A.; Liu F.; Kluetzman K.; Yang W.; Ding X. Generation and characterization of a novel *Cyp2a(4/5)bgs*-null mouse model. Drug Metab. Dispos. 2013, 41 (1), 132–140. 10.1124/dmd.112.048736. 23073733 PMC3533424

[ref56] Li L.; Megaraj V.; Wei Y.; Ding X. Identification of cytochrome P450 enzymes critical for lung tumorigenesis by the tobacco-specific carcinogen 4-(methylnitrosamino)-1-(3-pyridyl)-1-butanone (NNK): insights from a novel *Cyp2abfgs*-null mouse. Carcinogenesis 2014, 35 (11), 2584–2591. 10.1093/carcin/bgu182. 25173884 PMC4216058

[ref57] Uwimana E.; Maiers A.; Li X.; Lehmler H. J. Microsomal metabolism of prochiral polychlorinated biphenyls results in the enantioselective formation of chiral metabolites. Environ. Sci. Technol. 2017, 51 (3), 1820–1829. 10.1021/acs.est.6b05387. 28038482 PMC5300040

[ref58] Kania-Korwel I.; Duffel M. W.; Lehmler H.-J. Gas chromatographic analysis with chiral cyclodextrin phases reveals the enantioselective formation of hydroxylated polychlorinated biphenyls by rat liver microsomes. Environ. Sci. Technol. 2011, 45 (22), 9590–9596. 10.1021/es2014727. 21966948 PMC3216237

[ref59] Asher B. J.; D’Agostino L. A.; Way J. D.; Wong C. S.; Harynuk J. J. Comparison of peak integration methods for the determination of enantiomeric fraction in environmental samples. Chemosphere 2009, 75 (8), 1042–1048. 10.1016/j.chemosphere.2009.01.041. 19215960

[ref60] Wu X. N.; Lehmler H. J. Effects of thiol antioxidants on the atropselective oxidation of 2,2′,3,3′,6,6′-hexachlorobiphenyl (PCB 136) by rat liver microsomes. Environ. Sci. Pollut. Res. Int. 2016, 23 (3), 2081–2088. 10.1007/s11356-015-4987-4. 26155892 PMC4706823

[ref61] Davis J. C. Statistics and Data Analysis in Geology, 2nd ed.; Wiley: New York, NY, 1986.

[ref62] Wu X.; Zhai G.; Schnoor J. L.; Lehmler H.-J. Atropselective disposition of 2,2′,3,4′,6-pentachlorobiphenyl (PCB 91) and identification of its metabolites in mice with liver-specific deletion of cytochrome P450 reductase. Chem. Res. Toxicol. 2020, 33 (6), 1328–1338. 10.1021/acs.chemrestox.9b00255. 31403789 PMC7042073

[ref63] Kania-Korwel I.; Barnhart C. D.; Lein P. J.; Lehmler H.-J. Effect of pregnancy on the disposition of 2,2′,3,5′,6-pentachlorobiphenyl (PCB 95) atropisomers and their hydroxylated metabolites in female mice. Chem. Res. Toxicol. 2015, 28 (0), 1774–1783. 10.1021/acs.chemrestox.5b00241. 26271003 PMC4579038

[ref64] Kania-Korwel I.; Barnhart C. D.; Stamou M.; Truong K. M.; El-Komy M. H.; Lein P. J.; Veng-Pedersen P.; Lehmler H.-J. 2,2′,3,5′,6-Pentachlorobiphenyl (PCB 95) and its hydroxylated metabolites are enantiomerically enriched in female mice. Environ. Sci. Technol. 2012, 46 (20), 11393–401. 10.1021/es302810t. 22974126 PMC3475189

[ref65] Sundström G.; Jansson B. The metabolism of 2,2′,3,5′,6-pentachlorobiphenyl in rats, mice and quails. Chemosphere 1975, 4 (6), 361–370. 10.1016/0045-6535(75)90032-6.

[ref66] Stamou M.; Uwimana E.; Flannery B. M.; Kania-Korwel I.; Lehmler H. J.; Lein P. J. Subacute nicotine co-exposure has no effect on 2,2′,3,5′,6-pentachlorobiphenyl disposition but alters hepatic cytochrome P450 expression in the male rat. Toxicology 2015, 338, 59–68. 10.1016/j.tox.2015.10.002. 26463278 PMC4658283

[ref67] Schnellmann R. G.; Putnam C. W.; Sipes I. G. Metabolism of 2,2′,3,3′,6,6′-hexachlorobiphenyl and 2,2′,4,4′,5,5′-hexachlorobiphenyl by human hepatic microsomes. Biochem. Pharmacol. 1983, 32 (21), 3233–3239. 10.1016/0006-2952(83)90209-5. 6416258

[ref68] Wu X.; Kammerer A.; Lehmler H. J. Microsomal oxidation of 2,2’,3,3′,6,6’-hexachlorobiphenyl (PCB 136) results in species-dependent chiral signatures of the hydroxylated metabolites. Environ. Sci. Technol. 2014, 48 (4), 2436–44. 10.1021/es405433t. 24467194 PMC3983324

[ref69] Kania-Korwel I.; Vyas S. M.; Song Y.; Lehmler H.-J. Gas chromatographic separation of methoxylated polychlorinated biphenyl atropisomers. J. Chromatogr. A 2008, 1207 (1–2), 146–154. 10.1016/j.chroma.2008.08.044. 18760792 PMC2579784

[ref70] Asaoka Y.; Sakai H.; Sasaki J.; Goryo M.; Yanai T.; Masegi T.; Okada K. Changes in the gene expression and enzyme activity of hepatic cytochrome P450 in juvenile Sprague-Dawley rats. J. Vet. Med. Sci. 2010, 72 (4), 471–479. 10.1292/jvms.09-0397. 20032627

[ref71] Li X.; Wu X.; Kelly K. M.; Veng-Pedersen P.; Lehmler H.-J. Toxicokinetics of chiral PCB 136 and its hydroxylated metabolites in mice with a liver-specific deletion of cytochrome P450 reductase. Chem. Res. Toxicol. 2019, 32 (4), 727–736. 10.1021/acs.chemrestox.8b00389. 30729780 PMC6465157

[ref72] Lehmler H.-J.; Robertson L. W.; Garrison A. W.; Kodavanti P. R. S. Effects of PCB 84 enantiomers on [^3^H] phorbol ester binding in rat cerebellar granule cells and ^45^Ca^2+^-uptake in rat cerebellum. Toxicol. Lett. 2005, 156, 391–400. 10.1016/j.toxlet.2004.12.011. 15763638

[ref73] Ma C.; Zhai G.; Wu H.; Kania-Korwel I.; Lehmler H. J.; Schnoor J. L. Identification of a novel hydroxylated metabolite of 2,2′,3,5′,6-pentachlorobiphenyl formed in whole poplar plants. Environ. Sci. Pollut. Res. Int. 2016, 23 (3), 2089–2098. 10.1007/s11356-015-5939-8. 26676542 PMC4718877

[ref74] Wang H.; Donley K. M.; Keeney D. S.; Hoffman S. M. Organization and evolution of the Cyp2 gene cluster on mouse chromosome 7, and comparison with the syntenic human cluster. Environ. Health Perspect. 2003, 111 (15), 1835–42. 10.1289/ehp.6546. 14630516 PMC1241748

