# Supplementary material for: Probing the Role of CYP2 Enzymes in the Atropselective Metabolism of Polychlorinated Biphenyls Using Liver Microsomes from Transgenic Mouse Models
Source: Chem Res Toxicol. 2022 Dec 6;35(12):2310–23. doi: 10.1021/acs.chemrestox.2c00276 (PMC9957597; doi:10.1021/acs.chemrestox.2c00276)
Supplement: Supplementary file 1 — tx2c00276_si_001.pdf [file tx2c00276_si_001.pdf]

SUPPORTING INFORMATION

Probing the Role of CYP2 Enzymes in the Atropselective Metabolism of Polychlorinated Biphenyls Using Liver Microsomes from Transgenic Mouse Models

Hans-Joachim Lehmler,[†] Eric Uwimana,[†] Laura E. Dean,[†]
Nataliia Kovalchuk,[‡] Qing-Yu Zhang, [‡] Xinxin Ding,[‡]

[†] Interdisciplinary Graduate Program in Human Toxicology and Department of Occupational and Environmental Health, University of Iowa, Iowa City, IA 52242, United States. [‡] Department of Pharmacology & Toxicology, College of Pharmacy, University of Arizona, Tucson, AZ 85721, United States.

Corresponding Author:
Dr. Hans-Joachim Lehmler
The University of Iowa
Department of Occupational and Environmental Health
University of Iowa Research Park, B164 MTF
Iowa City, IA 52242-5000
Phone: (319) 335-4981
Fax: (319) 335-4290
e-mail: hans-joachim-lehmler@uiowa.edu

Number of pages: 19

Number of tables: 6

Number of figures: 6

Table of Content

Abbreviations S-4

Table S1. Intraday and inter-day variation of metabolism studies with racemic PCB 91 were assessed with pooled mouse liver microsomes (Xenotech, Lenexa, KS, USA). S-5

Table S2. Levels of hydroxylated PCB metabolites (ng/mg protein) formed from chiral PCBs incubated with mouse liver microsomes from *Cyp2abfgs*-null, *Cyp2a(4/5)bgs*-null, *Cyp2f2*-null, and wild-type (WT) mice. S-6

Table S3. Chiral capillary gas chromatography columns used in the enantioselective separation of PCBs and their metabolites. S-8

Table S4. Enantiomeric fraction, retention time, and resolution of racemic standards; and analysis temperatures of PCB and OH-PCB atropisomers determined using different enantioselective columns. S-8

Table S5. Limit of detection (ng) of PCB metabolites (as methylated derivatives). S-10

Table S6. Similarity coefficient cos θ comparing the hydroxylated metabolite profiles formed by different microsomal preparations. S-11

Figure S1. Representative gas chromatograms comparing the racemic standards (panels A1-A3) to 5-91 and 4-91 formed in microsomal incubations reveal the atropselective formation of E_1-4-91 and E_2-5-91 in incubations of racemic PCB 91 with mouse liver microsomes prepared from male or female WT mice (panels B1 & B2), and female *Cyp2a(4/5)bgs*-null mice (panel C). S-12

Figure S2. Representative gas chromatograms comparing the racemic standard to 3-100 formed in microsomal incubations reveal the atropselective formation of E_1-3-100 in incubations of racemic PCB 91 with mouse liver microsomes prepared from male *Cyp2a(4/5)bgs*-null and male or female WT mice. S-14

Figure S3. Representative gas chromatograms comparing the racemic standards (panels A1 & A2) to 4'-95 formed in microsomal incubations reveal the atropselective formation of E_1-4'-95 in incubations of racemic PCB 95 with mouse liver microsomes prepared from male or female *Cyp2a(4/5)bgs*-null mice (panels B1 & B2) and male or female WT mice (panels C1-C2). S-15

Figure S4. Representative gas chromatograms comparing the racemic standards (panels A1 & A2) to 5'-132 formed in microsomal incubations reveal the atropselective formation of E_2-5'-132 in incubations of racemic PCB 132 with mouse liver microsomes prepared from male or female *Cyp2a(4/5)bgs*-null mice (panels B1 & B2) and WT mice (panels C1 & C2). S-16

Figure S5. Representative gas chromatograms comparing the racemic standard (panels A1 & A2) to 3'-140 formed in microsomal incubations reveal the atropselective formation of E_2-3'-140 in incubations of racemic PCB 132 with mouse liver microsomes prepared from male *Cyp2a(4/5)bgs*-null mice (panel B) and female WT mice (panel C). S-17

Figure S6. Representative gas chromatograms comparing racemic standards of 4-136 (panels A1 & A2) and 5-136 (panels B1 & B2) to 4-136 and 5-136 formed in S-18

microsomal incubations reveal the atropselective formation of E_1-4-136 and E_2-5-136 in incubations of racemic PCB 136 with mouse liver microsomes prepared from male or female *Cyp2a(4/5)bgs*-null mice (panels C1 & C2 for 4-136 and panels D1 & D2 for 5-136), male or female *Cyp2f2*-null mice (panels E1 & E2 for 4-136 and panels F1 & F2 for 5-136), and male or female WT mice (panels G1a to G2b for 4-136 and panels H1a to H2b for 4-136).

References S-19

Abbreviations

3-100	2,2',4,4',6-pentachlorobiphenyl-3-ol
3-103	2,2',4,5',6-pentachlorobiphenyl-3-ol
3'-140	2,2',3,4,4',6'-hexachlorobiphenyl-3-ol
3-150	2,2',3',4,6,6'-hexachlorobiphenyl-3-ol
4-91	2,2',3,4',6-pentachlorobiphenyl-4-ol
4-95	2,2',3,5',6-pentachlorobiphenyl-4-ol
4'-95	2,2',3,5',6-pentachlorobiphenyl-4-ol
4'-132	2,2',3,3',4,6'-hexachlorobiphenyl-4'-ol
4-136	2,2',3,3',6,6'-hexachlorobiphenyl-4-ol
5-91	2,2',3,4',6-pentachlorobiphenyl-5-ol
5-95	2,2',3,5',6-pentachlorobiphenyl-5-ol
5'-132	2,2',3,3',4,6'-hexachlorobiphenyl-5'-ol
5-136	2,2',3,3',6,6'-hexachlorobiphenyl-5-ol
4,5-91	4,5-dihydroxy-2,2',3,4',6-pentachlorobiphenyl
4,5-95	4,5-dihydroxy-2,2',3,5',6-pentachlorobiphenyl
4',5'-132	4',5'-dihydroxy-2,2',3,3',4,6'-hexachlorobiphenyl
4,5-136	4,5-dihydroxy-2,2',3,3',6,6'-hexachlorobiphenyl
BDM	ChiralDex B-DM
CB	Cyclosil-B
CD	CP Chirasil-Dex CB
E_1	Atropisomer eluting first on the respective chiral column
E_2	Atropisomer eluting second on the respective chiral column
EF	Enantiomeric fraction
GTA	ChiralDex G-TA
NADPH	Nicotine adenine dinucleotide phosphate (reduced)
nd	Not determined
nr	Not resolved
OH-PCB	Hydroxylated polychlorinated biphenyl metabolite
PCB	Polychlorinated biphenyl
PCB 91	2,2',3,4',6-Pentachlorobiphenyl
PCB 95	2,2',3,5',6-pentachlorobiphenyl
PCB 117	2,3,4',5,6-pentachlorobiphenyl (recovery standard),
PCB 132	2,2',3,3',4,6'-hexachlorobiphenyl
PCB 136	2,2',3,3',6,6'-hexachlorobiphenyl
PCB 204	2,2',3,4,4',5,6,6'-octachlorobiphenyl (internal standard)
Rs	Resolution
SD	Standard deviation
WT	Wild-type

Table S1. Intraday and inter-day variation of metabolism studies with racemic PCB 91 were assessed with pooled mouse liver microsomes (Xenotech, Lenexa, KS, USA). Levels of PCB metabolite (ng/mg microsomal protein) are expressed as mean ± SD (relative standard deviation), n = 3.[a]

Incubation	1,2 shift product	5-OH-PCB	4-OH-PCB	Σ OH-PCBs
Day 1	29 ± 5 (19)	63 ± 9 (14)	31 ± 5 (16)	123 ± 18 (16)
Day 2	27 ± 3 (9)	61 ± 5 (8)	29 ± 3 (9)	117 ± 11 (8)
Day 3	31 ± 1 (2)	67 ± 1 (2)	33 ± 0.3 (1)	131 ± 0.2 (0.2)
Day 4	35 ± 5 (16)	55 ± 6 (10)	28 ± 4 (12)	120 ± 14 (12)
Interday	31 ± 2 (7)	62 ± 3 (5)	30 ± 2 (6)	123 ± 8 (6)

[a] Metabolism studies with pooled mouse liver microsomes were performed using the following incubation conditions: 50 µM PCB; 30-minute incubation at 37 ºC; 0.1 mg/mL microsomal protein content; and 1 mM NADPH.

Table S2. Levels of hydroxylated PCB metabolites (ng/mg protein) formed from chiral PCBs incubated with mouse liver microsomes from *Cyp2a(4/5)bgs*-null, *Cyp2f2*-null, and WT mice. Data are expressed as mean ± SD, n = 3[a].

**Mouse model**	**male mice**					**female mice**				
	**1,2 shift product**	**5-OH-PCB**	**4-OH-PCB**	**4'-OH-PCB 95**	**Σ OH-PCBs**	**1,2 shift product**	**5-OH-PCB**	**4-OH-PCB**	**4'-OH-PCB 95**	**Σ OH-PCBs**
**PCB 91**										
Cyp2a(4/5)bgs-null	20.7 ± 1.5[c]	18.7 ± 0.6[b]	24.0 ± 1.0[bc]		63.3 ± 2.5[bc]	25.0 ± 1.0	25.3 ± 0.6[bcd]	40.7 ± 2.1[bcd]		90.7 ± 3.8[d]
Cyp2f2-null	6.2 ± 0.2[b]	21.0 ± 0.0[b]	41.0 ± 0.0[b]		68.0 ± 0.0	13.3 ± 0.6[b]	41.3 ± 3.2[bd]	50.0 ± 2.6[bd]		106.3 ± 7.1[d]
WT	30.8 ± 7.1	28.5 ± 2.8	28.7 ± 2.2		88.0 ± 5.8	25.7 ± 7.6	52.2 ± 3.0[d]	24.7 ± 3.3[d]		102.3 ± 12.4[d]
**PCB 95**										
Cyp2a(4/5)bgs-null	0.5 ± 0.1[bc]	43.6 ± 5.6[c]	15.1 ± 2.3[bc]	16.0 ± 2.4	75.4 ± 10.6[b]	0.6 ± 0.1	70.3 ± 3.1[bd]	18.0 ± 0.0[c]	17.7 ± 0.6[bc]	107.0 ± 4.0[d]
Cyp2f2-null	0.8 ± 0.1[b]	53.0 ± 3.0	27.3 ± 1.5[b]	15.6 ± 1.1	96.7 ± 5.6[b]	1.1 ± 0.1	67.3 ± 4.0[bd]	29.3 ± 2.1[b]	29.0 ± 1.7[bd]	126.7 ± 8.5[bd]
WT	1.0 ± 0.1	50.8 ± 3.3	21.3 ± 2.1	17.8 ± 1.7	93.2 ± 9.4	0.8 ± 0.3	58.0 ± 5.4	19.7 ± 2.7	21.0 ± 0.9[d]	100.0 ± 9.9
**PCB 132**										
Cyp2a(4/5)bgs-null	6.4 ± 0.7[c]	9.3 ± 0.4[b]	14.1 ± 0.9[c]		29.2 ± 1.9[bc]	6.5 ± 2.8	15.67 ± 5.5[bc]	17.7 ± 6.4		40.0 ± 14.7[b]
Cyp2f2-null	2.5 ± 0.3[b]	16.3 ± 0.6[b]	21.6 ± 2.9[b]		40.3 ± 4.0	3.8 ± 0.3	25.7 ± 2.1[bd]	16.7 ± 1.2		46.3 ± 3.2[b]
WT	5.7 ± 0.5	21.2 ± 3.5	15.4 ± 0.5		43.0 ± 3.2	6.2 ± 0.3	70.0 ± 2.0[d]	18.2 ± 1.1		94.8 ± 0.8[d]
**PCB 136**										
Cyp2a(4/5)bgs-null	3.0 ± 0.3	38.2 ± 3.6[bc]	27.1 ± 2.9[b]		69.7 ± 9.3[b]	3.4 ± 0.3	79.0 ± 4.3[d]	31.0 ± 1.7		117.3 ± 5.9
Cyp2f2-null	4.1 ± 0.1	45.3 ± 1.2[b]	23.3 ± 1.2[b]		78.7 ± 1.5[b]	3.8 ± 0.4	80.3 ± 7.3[d]	37.3 ± 4.7[d]		133.7 ± 11.9[d]
WT	4.0 ± 0.8	60.7 ± 2.9	34.7 ± 1.6		108.5 ± 10.6	3.3 ± 0.6	86.0 ± 8.7[d]	32.0 ± 3.2		127.2 ± 14.9[d]

[a] Metabolism studies with mouse liver microsomal preparations from *Cyp2a(4/5)bgs*-null, *Cyp2f2*-null, and WT mice were performed using the following incubation conditions: 50 µM PCB; 30-minute incubation at 37 ºC; 0.1 mg/mL microsomal protein content; and 1 mM NADPH. Microsomes were prepared in-house from C57BL/6 WT mice and knockout mice on the same genetic background, as described (Wei et al., 2012).
[b] Levels of metabolite(s) are significantly different in *Cyp2a(4/5)bgs*-null or *Cyp2f2*-null compared to WT ($p \leq 0.05$)
[c] Levels of metabolite(s) are significantly different in *Cyp2a(4/5)bgs*-null compared to *Cyp2f2*-null ($p \leq 0.05$)
[d] Levels of metabolite(s) are significantly different in females compared to males ($p \leq 0.05$)

Table S3. Chiral capillary gas chromatography columns used in the enantioselective separation of PCBs and their metabolites.[a]

Column name	Abbreviation	Supplier	Colum length × inner diameter × film thickness	Chiral stationary phase	Cyclodextrin type
ChiralDex B-DM	BDM	Supelco Analytical St. Louis, MO	30 m × 250 µm × 0.12 µm	2,3-di-*O*-methyl-6-*tert*-butyl-silyl-	β-cyclodextrin
CP Chirasil-Dex CB	CD	Varian (Agilent) Santa Clara, CA	25 m × 250 µm × 0.12 µm	2,3,6-tri-*O*-methyl-	β-cyclodextrin
Cyclosil-B	CB	Agilent Santa Clara, CA	30 m × 250 µm × 0.25 µm	30% hepatkis (2,3-di-*O*-methyl-6-*O*-t-butyl dimethyl-silyl)-	β-cyclodextrin
ChiralDex G-TA	GTA	Supelco Analytical St. Louis, MO	30 m × 250 µm × 0.12 µm	2,6-di-*O*-pentyl-3-trifluoroacetyl-	γ-cyclodextrin

[a] Information as provided by the column manufacturer. The use of these columns for enantioselective separations of the parent PCBs and their hydroxylated metabolites (as methylated derivatives) has been reported previously (Kania-Korwel et al., 2011; Kania-Korwel et al., 2008).

Table S4. Enantiomeric fraction, retention time, and resolution of racemic standards; and analysis temperatures of PCB and OH-PCB atropisomers determined using different enantioselective columns.

**Parent PCB**	**Racemic standard EF**	**Tested columns: Resolution,[a] (retention time [min],[b] isothermal temperature *x*[c])**			
		**BDM**	**CD**	**CB**	**GTA**
**PCB 91**	0.50 ± 0.002 (n=5)	1.12 (125, 135°C)	-	-	-
**3-100**	0.49 ± 0.01 (n=7)	-	-	-	0.76 (278, 140°C)
**5-91**	0.50 ± 0.002 (n=4)	4.50 (445, 135°C)	-	-	-
**4-91**	0.49 ± 0.01 (n=4)	0.94 (430, 135°C)	-	-	-
**PCB 95**	0.50 ± 0.01 (n=8)	1.09 (89, 139°C)	-	-	-
**3-103**	0.47 ± 0.03 (n=8)	0.55 (157, 139°C,)	-	-	-
**4'-95**	0.49 ± 0.01 (n=8)	1.65 (324, 139°C)	-	-	-
**PCB 132**	0.50 ± 0.001 (n=5)	1.08 (81.1, 160°C)	-	-	-
**3'-140**	0.50 ± 0.002 (n=4)	-	-	-	1.09 (402, 150°C)
**5'-132**	0.50 ± 0.01 (n=8)	2.02 (221, 160°C)	-	-	-
**PCB 136**	0.49 ± 0.01 (CD, n=6) 0.50 ± 0.00 (CB n=4)	-	1.02 (182, 140°C)	1.07 (107, 160°C)	-
**3-150**	0.50 ± 0.002 (CD, n=6) 0.50 ± 0.01 (CD, n=4)	-	0.88 (295, 140°C)	0.63 (172, 160°C)	-
**5-136**	0.50 ± 0.001 (CD, n=6)	-	0.86 (524, 140°C)	-	-
**4-136**	0.50 ± 0.003 (CD, n=6) 0.50 ± 0.00 (CB, n=4)	-	1.05 (605, 140°C)	0.87 (360, 160°C)	-

[a] The resolution (Rs) was calculated using the formula $Rs = (t_{R2} - t_{R1})/0.5(BW_1 + BW_2)$, where t_{R2} and t_{R1} are the retention times of the first and second eluting atropisomer, and BW_1 and BW_2 are the baseline width of the first and second eluting atropisomer (Kania-Korwel et al., 2011).
[b] The retention time of the first eluting atropisomer (min).
[c] Temperature program: initial temperature, 50 °C for 1 min; ramped to *x* °C at 10 °C/min (x = 135 to 160 °C); hold for up to 650 min; then to final 180 to 220 °C for 10 to 40 min depending

on column and analyte(s); *x* is the temperature of the isothermal segment of the temperature program. Injector and detector temperatures were 250 °C. Helium flow was maintained at 3 mL/min.
- No data available.
Column abbreviations: BDM - Chiral-Dex B-DM; CD - Chirasil-Dex (CD); CB - Cyclosil-B; GTA - Chiral-Dex G-TA, see Table S7 for additional information.

Table S5. Limit of detection (ng) of PCB metabolites (as methylated derivatives).

**Parent PCB**	**1,2 shift product**	**5-OH-PCB**	**4-OH-PCB**	**4'-OH-95**
PCB 91	0.06	0.82	0.29	NA
PCB 95	0.04	0.25	0.89	0.98
PCB 132	0.07	0.14	0.30	NA
PCB 136	0.26	0.13	0.09	NA

NA, not applicable

Table S6. Similarity coefficient cos θ comparing the hydroxylated metabolite profiles formed by different microsomal preparations.

**Parent PCB**	**Male**		**Female**		**Male vs. female**	
	***Cyp2f2*-null vs. WT**	***Cyp2a(4/5)bgs*-null vs. WT**	***Cyp2f2*-null vs. WT**	***Cyp2a(4/5)bgs*-null vs. WT**	**WT**	***Cyp2f2*-null**
PCB 91	0.83	0.99	0.89	0.87	0.93	0.90
PCB 95	1.00	1.00	1.00	0.99	1.00	0.99
PCB 132	0.94	0.94	0.95	0.82	0.92	0.99
PCB 136	1.00	0.99	0.99	1.00	0.98	1.00

The cos θ ranges from 0 to 1, where a value of 0 indicates completely different profiles and a value of 1 indicates identical profiles.

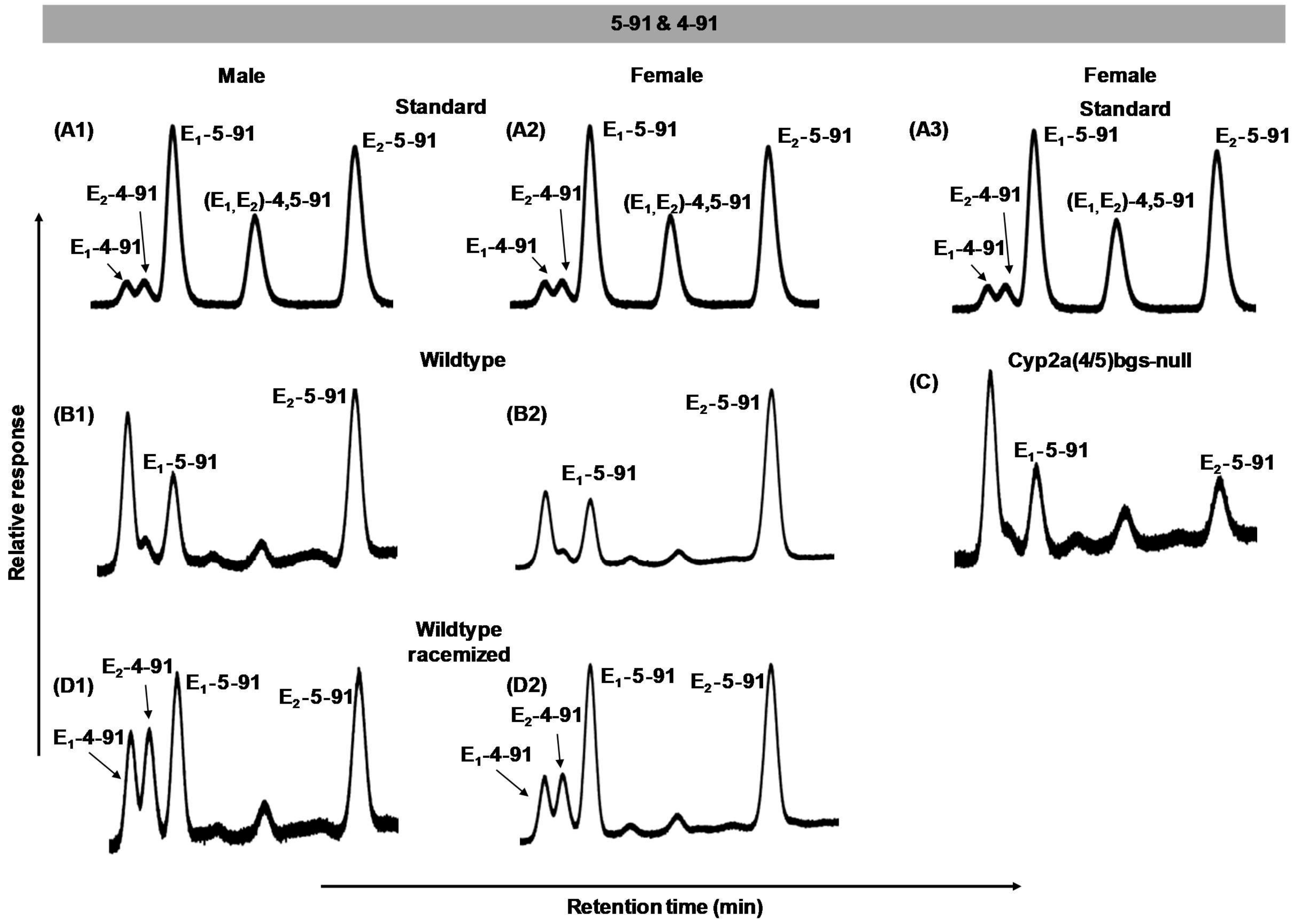

Figure S1. Representative gas chromatograms comparing the racemic standards (panels A1-A3) to 5-91 and 4-91 formed in microsomal incubations reveal the atropselective formation of E_1-4-91 and E_2-5-91 in incubations of racemic PCB 91 with mouse liver microsomes

prepared from male or female wild-type mice (panels B1 & B2), and female *Cyp2a(4/5)bgs*-null mice (panel C). The identification of the 5-91 and 4-91 atropisomers was verified for selected experiments using microsomes from male or female wild-type microsomes (panels D1 & D2) by reanalyzing the extracts after racemizing them at 300 °C, as described (Uwimana et al., 2017). Microsomal incubations with 50 μM racemic PCB 91 were carried out at 37 °C for 30 min. The 4-91 and 5-91 atropisomers were separated on a BDM column as methylated derivatives.

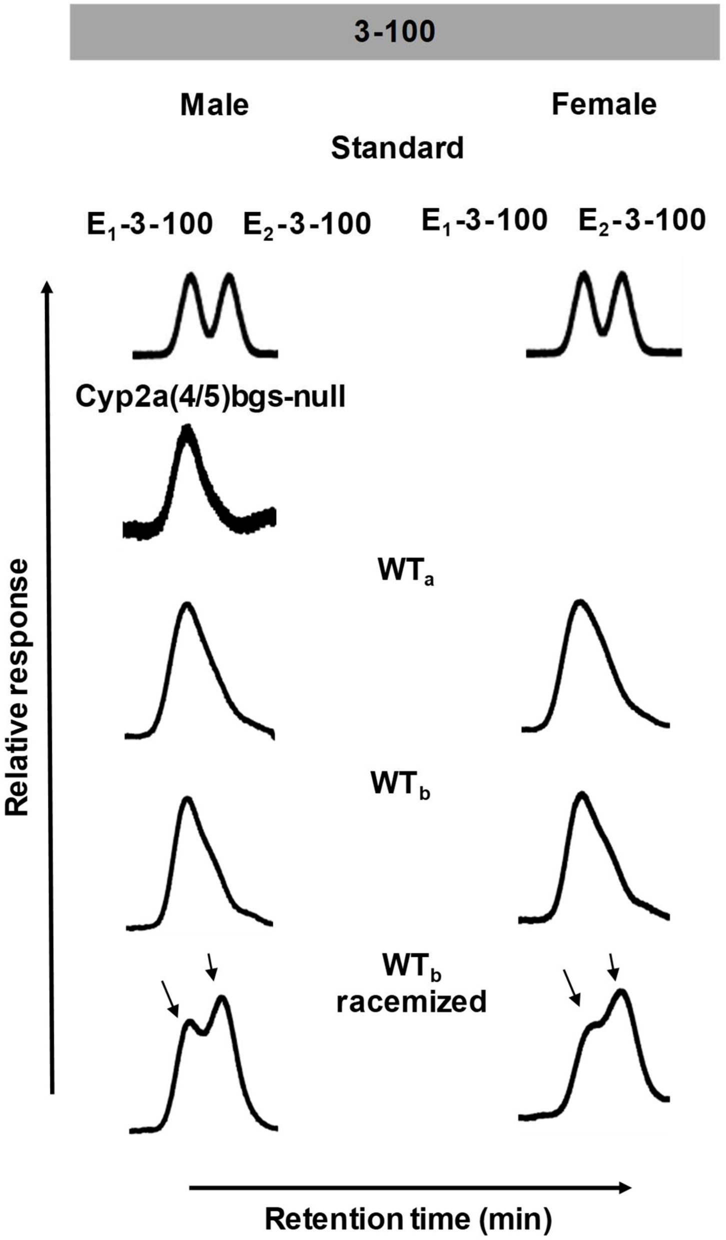

Figure S2. Representative gas chromatograms comparing the racemic standard to 3-100 formed in microsomal incubations reveal the atropselective formation of E_1-3-100 in incubations of racemic PCB 91 with mouse liver microsomes prepared from male *Cyp2a(4/5)bgs*-null and male or female WT mice. Representative extracts from incubations with male or female WT microsomes were racemized at 300 °C and reanalyzed to confirm the identification of the 3-100 atropisomers (Uwimana et al., 2017). Microsomal incubations with 50 µM racemic PCB 91 were carried out at 37 °C for 30 min. The atropisomers of 3-100 were separated on a GTA column as methylated derivatives. WT_a and WT_b are chromatograms from different experiments with microsomes from WT mice.

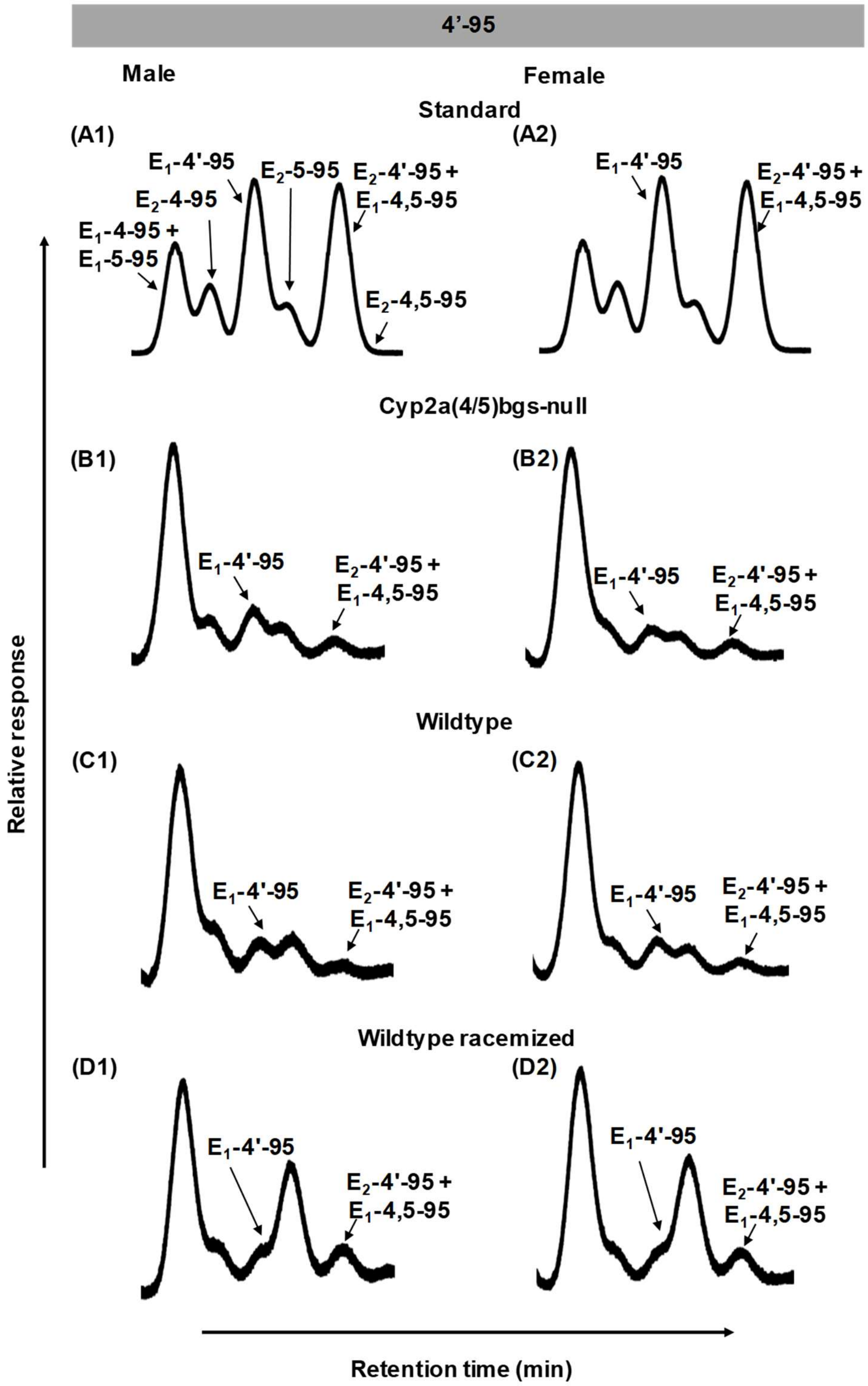

Figure S3. Representative gas chromatograms comparing the racemic standards (panels A1 & A2) to 4'-95 formed in microsomal incubations reveal the atropselective formation of E_1-4'-95 in incubations of racemic PCB 95 with mouse liver microsomes prepared from male or female *Cyp2a(4/5)bgs*-null mice (panels B1 & B2) and male or female wild-type mice (panels C1-C2). The identification of the 4'-95 atropisomers was verified for selected experiments using microsomes from male or female wild-type microsomes (panels D1 & D2) by reanalyzing the

extracts after racemizing them at 300 °C, as described (Uwimana et al., 2017). Microsomal incubations with 50 µM racemic PCB 95 were carried out at 37 °C for 30 min. The atropisomers of 5-95, 4'-95, and 4-95 were separated on a BDM column as methylated derivatives.

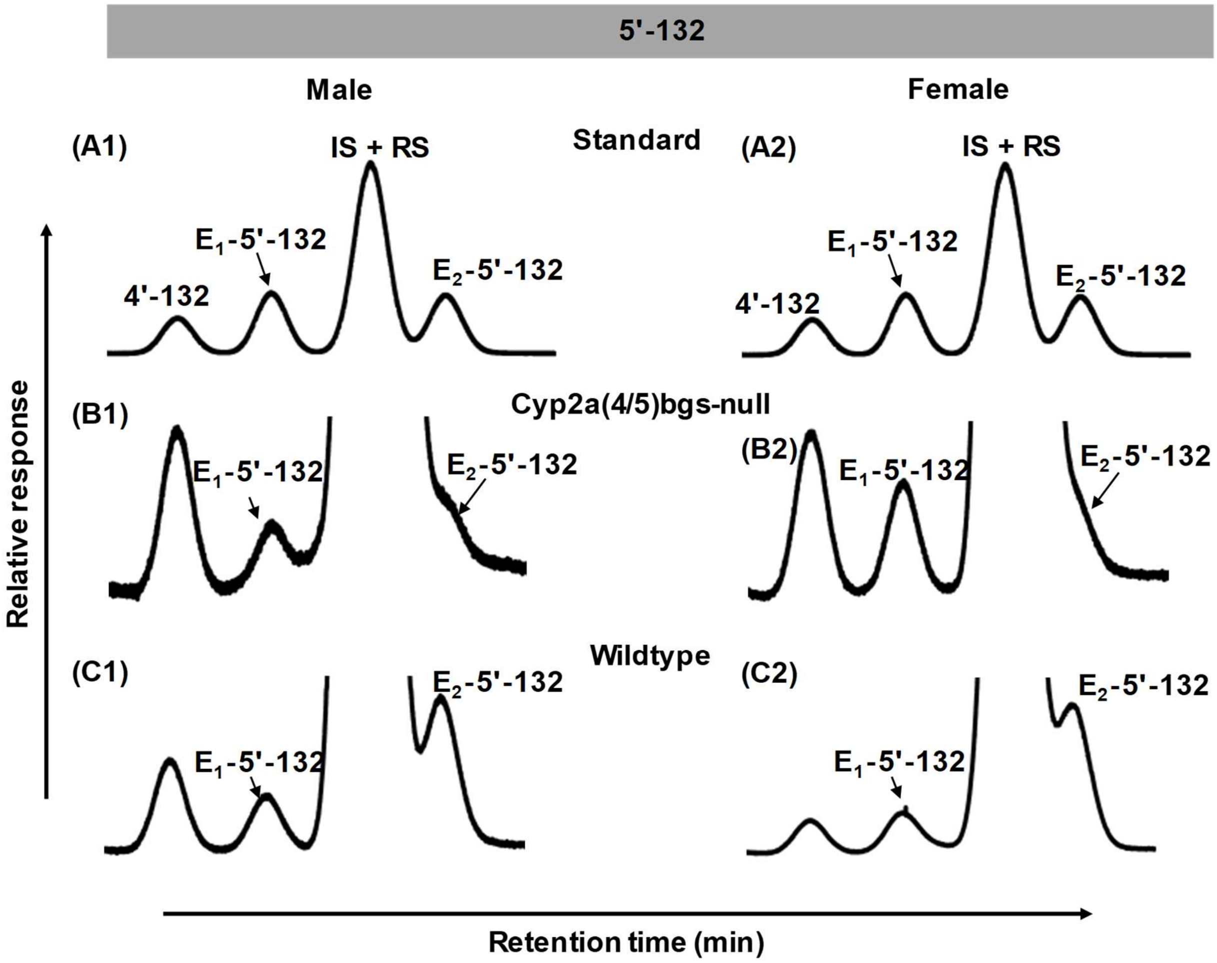

Figure S4. Representative gas chromatograms comparing the racemic standards (panels A1 & A2) to 5'-132 formed in microsomal incubations reveal the atropselective formation of E_2-5'-132 in incubations of racemic PCB 132 with mouse liver microsomes prepared from male or female *Cyp2a(4/5)bgs*-null mice (panels B1 & B2) and wild-type mice (panels C1 & C2). Microsomal incubations with 50 µM racemic PCB 132 were carried out at 37 °C for 30 min. The atropisomers of 5'-132 were separated on a BDM column as methylated derivatives.

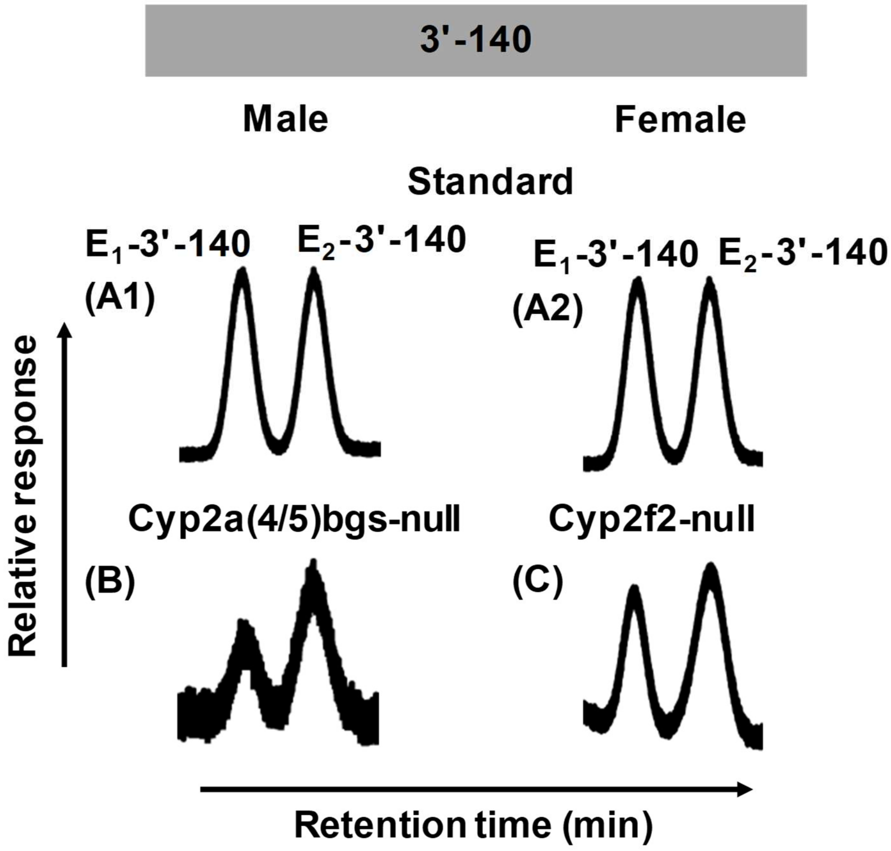

Figure S5. Representative gas chromatograms comparing the racemic standard (panels A1 & A2) to 3'-140 formed in microsomal incubations reveal the atropselective formation of E_2-3'-140 in incubations of racemic PCB 132 with mouse liver microsomes prepared from male *Cyp2a(4/5)bgs*-null mice (panel B) and female *Cyp2f2*-null mice (panel C). Microsomal incubations with 50 μM racemic PCB 132 were carried out at 37 °C for 30 min. The atropisomers of 3'-140 were separated on a GTA column as methylated derivatives.

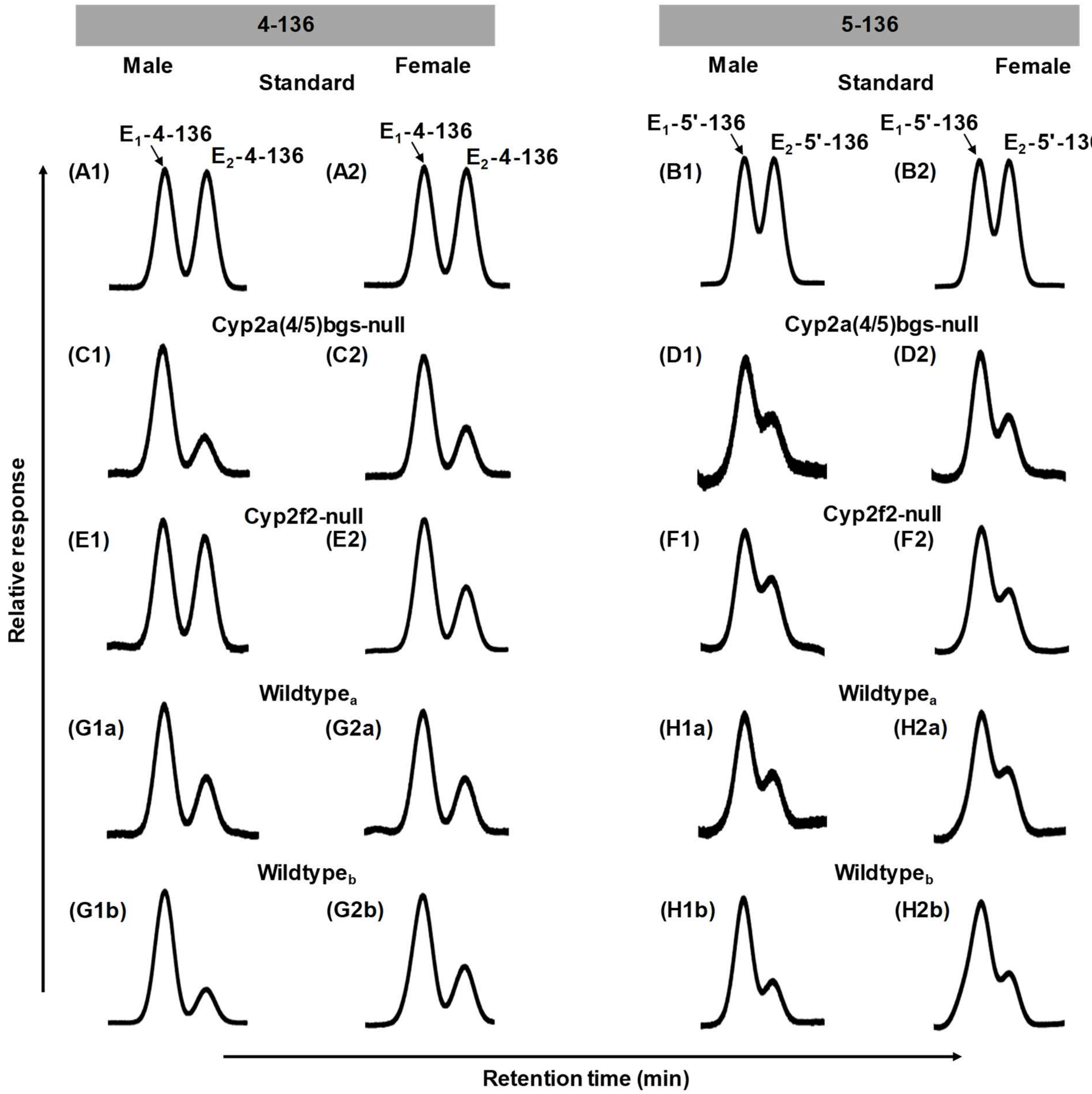

Figure S6. Representative gas chromatograms comparing racemic standards of 4-136 (panels A1 & A2) and 5-136 (panels B1 & B2) to 4-136 and 5-136 formed in microsomal incubations reveal the atropselective formation of E_1-4-136 and E_2-5-136 in incubations of racemic PCB 136 with mouse liver microsomes prepared from male or female *Cyp2a(4/5)bgs*-null mice (panels C1 & C2 for 4-136 and panels D1 & D2 for 5-136), male or female *Cyp2f2*-null mice (panels E1 & E2 for 4-136 and panels F1 & F2 for 5-136), and male or female wild-type mice (panels G1a to G2b for 4-136 and panels H1a to H2b for 4-136). Microsomal incubations with 50 µM racemic PCB 136 were carried out at 37 °C for 30 min. The atropisomers of 4-136 and 5-136 were separated on CD and CB columns as methylated derivatives. Wildtype$_a$ and wildtype$_b$ are chromatograms from different experiments with microsomes from wild-type mice.

References

Kania-Korwel, I., Duffel, M.W., Lehmler, H.J., 2011. Gas chromatographic analysis with chiral cyclodextrin phases reveals the enantioselective formation of hydroxylated polychlorinated biphenyls by rat liver microsomes. Environ. Sci. Technol. 45, 9590-9596. https://doi.org/10.1021/es2014727.

Kania-Korwel, I., Vyas, S.M., Song, Y., Lehmler, H.J., 2008. Gas chromatographic separation of methoxylated polychlorinated biphenyl atropisomers. J. Chromatogr. A 1207, 146-154. https://doi.org/10.1016/j.chroma.2008.08.044.

Uwimana, E., Maiers, A., Li, X., Lehmler, H.J., 2017. Microsomal metabolism of prochiral polychlorinated biphenyls results in the enantioselective formation of chiral metabolites. Environ. Sci. Technol. 51, 1820-1829. https://doi.org/10.1021/acs.est.6b05387.

Wei, Y., Wu, H., Li, L., Liu, Z., Zhou, X., Zhang, Q.Y., Weng, Y., D'Agostino, J., Ling, G., Zhang, X., Kluetzman, K., Yao, Y., Ding, X., 2012. Generation and characterization of a CYP2A13/2B6/2F1-transgenic mouse model. Drug Metab. Dispos. 40, 1144-1150. https://doi.org/10.1124/dmd.112.044826.
